# Leveraging Data from a Provincial Electronic Immunization Registry to Analyze Immunization Coverage, Timeliness, and Defaulters Among 8.8 Million Children from the 2018 to 2023 Birth Cohorts in Sindh Province, Pakistan

**DOI:** 10.3390/vaccines12121327

**Published:** 2024-11-26

**Authors:** Fatima Miraj, Sundus Iftikhar, Muhammad Siddique, Vijay Kumar Dharma, Mubarak Taighoon Shah, Danya Arif Siddiqi, Subhash Chandir

**Affiliations:** 1 Maternal & Child Health, IRD Pakistan, Karachi 75190, Pakistan; 2 IRD Global, 16 Raffles Quay, Singapore 049145, Singapore; 3 Department of Infectious Disease Epidemiology and Dynamics, London School of Hygiene & Tropical Medicine, Keppel St., London WC1E 7HT, UK; 4 Johns Hopkins Bloomberg School of Public Health, Baltimore, MD 21205, USA

**Keywords:** full immunization coverage, immunization defaulter, vaccine timeliness, antigen-wise coverage, immunization outreach activities, COVID-19 pandemic, 2022 floods, measles, BCG, electronic immunization registry

## Abstract

Background/Objectives: Full immunization coverage in Pakistan remains suboptimal at 66%. An in-depth assessment is needed to understand the long-term trends in immunization and identify the extent of defaulters and associated risk factors of them being left uncovered by the immunization system. Methods: We conducted a 5-year analysis using the Government’s Provincial Electronic Immunization Registry data for the 2018–2023 birth cohorts in Sindh province. We analyzed 8,792,392 child-level immunization records from 1 January 2018 to 31 May 2024 to examine trends in immunization coverage, timeliness, defaulter rates, and associated risk factors; Results: Our findings indicate gradual improvements in immunization coverage, with full immunization rates increasing by 23.2% (from 47.5% to 70.7%) from 2018 to 2022. While timeliness declined from 2018 to 2021, it recovered in 2022 and 2023. Over the 5-year study period, >90% of children defaulted on vaccinations, with 34.8% fully covered and 9.1% uncovered. Children from urban areas (OR = 1.54; 95% CI = 1.52, 1.56; *p*-value < 0.001) and those enrolled through fixed immunization sites (OR = 2.11; 95% CI = 2.08, 2.15; *p*-value < 0.001) and mobile immunization vans (OR = 1.13; 95% CI = 1.13, 1.77; *p*-value = 0.003) were at higher risk of being uncovered defaulters. Conclusions: This study demonstrates improvements in immunization coverage in Sindh while highlighting the challenge of low timeliness and high default rates. Our findings provide insights to strengthen immunization access and timeliness, particularly in high-default areas, and can guide policies in similar low-income settings for more equitable and comprehensive immunization coverage.

## 1. Introduction

Routine immunization saves up to 4 million lives annually and averts 230 disability-adjusted life years (DALYs) for every 1000 under-five children vaccinated [1]. Since the launch of the Expanded Programme on Immunization (EPI) in 1974, coverage for three doses of the diphtheria, tetanus, and pertussis vaccine (DTP3) has increased from under 5% to 84% in 2023 [2,3]. Despite concerted efforts, progress to achieve universal and equitable coverage has been uneven, and immunization levels have plateaued in recent years [4]. Inequities in access to basic vaccines persist, with more than half of under- and never-immunized children residing in just ten low- and middle-income countries (LMICs) [4]. Initiatives like the Immunization Agenda 2030 and the Gavi 5.0 strategy have, therefore, been set forth with the aim of reaching 90% coverage of essential vaccines, reducing the number of under- and never-immunized children, and ensuring equitable vaccination access for all [5,6]. However, global instability and disruptions, including the COVID-19 pandemic, have severely impacted routine immunization efforts, widening the vaccination divide and complicating progress toward achieving global targets [7,8]. Amid these rapidly changing global dynamics, tracking trends in immunization coverage, timeliness, and related indicators is critical for identifying vulnerable populations and guiding policy interventions that can close coverage gaps and ensure equitable access to vaccines.

Pakistan is among the ten LMICs where over 60% of the world’s 21 million under- and never-immunized children reside [9]. Since the launch of the EPI in 1978, vaccination services have been provided free of cost in Pakistan, leading to an increase in full immunization coverage rates from nearly 40% to 66% [10,11]. However, despite these gains, the overall burden of VPDs is still high, and routine immunization coverage remains well below the national target of 90%. Pakistan also remains one of the only two polio-endemic countries, with 20 cases of wild poliovirus 1 reported in 2022 and six in 2023 [12,13]. Significant regional disparities result in inconsistent vaccination coverage, with vaccination rates varying across provinces, from 29% in Balochistan to 80% in Punjab [14]. A major challenge contributing to low immunization rates across the country is the high number of defaulters—children not adhering to the vaccination schedule. While 88% of children start their vaccination schedule, only 73% have been reported to complete it [14]. Recent external shocks and health emergencies, such as the COVID-19 pandemic and the 2022 floods, have further disrupted vaccination efforts, increasing the number of defaulters and leaving thousands of children at risk of dying because of vaccine-preventable causes [15].

While the issue of vaccination coverage in Pakistan is well-documented, most of the research is outdated; relies on secondary survey data; often focuses on national aggregates, which can obscure regional disparities; and fails to capture comprehensive child-level analyses, particularly over a multi-year period [16,17,18,19]. Some studies examine the impact of recent events such as COVID-19 and natural disasters on routine immunization [15,20], but they lack comprehensive, long-term analyses of how these disruptions have affected vaccination rates and whether coverage has recovered. Most of the literature also has only examined coverage and timeliness in isolation, without exploring deeper issues, such as defaulter rates, recovery of defaulters, and the specific risk factors contributing to defaulters remaining uncovered. As immunization remains a high priority for the EPI and the government, there is a need for an updated assessment to understand how the immunization landscape has evolved over the years, identifying children who have been consistently left behind and examining the impact of disruptions like the COVID-19 pandemic and the 2022 flooding. Our study addresses these gaps and provides a timely, comprehensive analysis of vaccination trends and defaulter rates. Such an assessment will be crucial for developing targeted, evidence-based strategies to improve vaccine delivery and system resilience, ensuring that all children receive the necessary vaccinations and that immunization programs are responsive to evolving needs and challenges.

We leveraged the Government of Sindh’s Provincial Electronic Immunization Registry data to conduct a comprehensive 5-year analysis of child-level longitudinal immunization records, focusing on birth cohorts from 2018 to 2023 in Sindh province, Pakistan. We examined trends in immunization coverage and timeliness for children born in the last five years to understand the progress in immunizations, and we conducted an in-depth analysis of defaulter children to delineate their socio-demographic characteristics, coverage rates in the context of external shocks, and risk factors associated with missed vaccinations.

## 2. Materials and Methods

### 2.1. Population

Sindh, the second most populous province located in Southern Pakistan, has a population of 55.7 million people and contributes nearly 30% to the country’s GDP [21,22]. Administratively, the province is divided into six divisions, which are further subdivided into 30 districts and 1123 union councils (UCs), which are the smallest administrative units [23]. The literacy rate in Sindh stands at 58%, and nearly half of the province’s population live in rural areas, of which 37% live below the poverty line (earning less than USD 2.15 per day) [24,25]. The under-five mortality rate in Sindh is 46 per 1000 live births, while the infant mortality rate is 39 per 1000 live births [26]. Sindh has an annual birth cohort of 1.8 million, with an immunization coverage rate well below the national average, at only 44% [26]. Vaccinations are primarily administered through three methods, namely fixed immunization sites, which are permanent healthcare facilities; routine outreach, where healthcare teams travel to communities within a day’s reach of the immunization center; and enhanced outreach activities (EOAs), which are a series of intensive immunization sessions targeting geographic areas beyond the routine outreach radius, particularly those with low coverage [27].

### 2.2. External Natural Shocks Impacting Immunization in Sindh

Over the past five years, several external shocks have affected routine immunization services in the Sindh province, Pakistan. The first confirmed case of COVID-19 in Pakistan was reported in Karachi, Sindh’s largest city, on 26 February 2020, followed by the first recorded death in the province on 20 March 2020 [28]. The pandemic led to various lockdown measures, beginning with a nationwide lockdown in March 2020 and eventually transitioning to a “smart lockdown” strategy targeting hotspot areas [29]. The pandemic was officially declared no longer a public health emergency as of 5 May 2023 [30].

In July 2022, Sindh was again severely affected by catastrophic floods caused by unprecedented monsoon rains, which displaced millions, destroyed infrastructure, and caused significant agricultural losses [31]. Both the COVID-19 pandemic and intense flooding disrupted health activities, particularly immunization efforts, and contributed to the outbreak of other VPDs, such as measles, polio, and diarrhea [15].

### 2.3. Data Source

We used immunization records from the Government of Sindh’s Electronic Immunization Registry (SEIR aka Zindagi Mehfooz (Safe Life) Electronic Immunization Registry (ZM-EIR)). The ZM-EIR utilizes Android technology to manage immunization procedures, enabling vaccinators to use smartphones to enroll children aged 0–23 months at their first vaccination through the registry and to follow up and track subsequent immunization events. The ZM-EIR collects comprehensive data, including children’s demographics, immunization details, the health facility, vaccinator information, and the geolocation of vaccinations. It tracks each child’s record using a unique identifier and monitors vaccinators’ performances and system usage compliance. Initially piloted in 2012, the ZM-EIR was scaled up across Sindh province in October 2017 as the Sindh Electronic Immunization Registry. As of May 2024, the ZM-EIR is being used by 4040 vaccinators (including 14.2% females) working at 2022 immunization centers and has enrolled over 10.6 million children and 4.3 million women, recording over 132 million individual-level immunization events.

### 2.4. Vaccination Schedule

Pakistan’s routine EPI immunization schedule includes six visits and covers 12 VPDs [10]. The vaccination schedule is as follows. The BCG (Bacille Calmette-Guérin) and oral polio vaccine (OPV-0) are administered at birth. At 6 weeks of age, the first doses of the pentavalent vaccine (DPT, HepB, and Hib), the pneumococcal conjugate vaccine (PCV-1), and the oral polio vaccine (OPV-1) are given. This is followed by the second doses of these vaccines at 10 weeks (Penta-2, PCV2, and OPV-2), and the third doses at 14 weeks (Penta3, PCV3, OPV-3), along with the first dose of inactivated polio vaccine (IPV-1) at 14 weeks. Additionally, the schedule includes two doses of rotavirus vaccine at 6 and 10 weeks (Rota-1 and Rota-2). The second dose of inactivated polio vaccine (IPV-2) is administered at 9 months, alongside the first dose of the measles–rubella vaccine (Measles-1). At 15 months, the second doses of the measles–rubella vaccine (Measles-2) and the typhoid conjugate vaccine (TCV) are given. The typhoid conjugate vaccine (TCV) was introduced into the routine immunization schedule on 1 January 2020, while the second dose of IPV was added on 3 May 2021.

### 2.5. Study Design and Procedure

**Figure 1:**
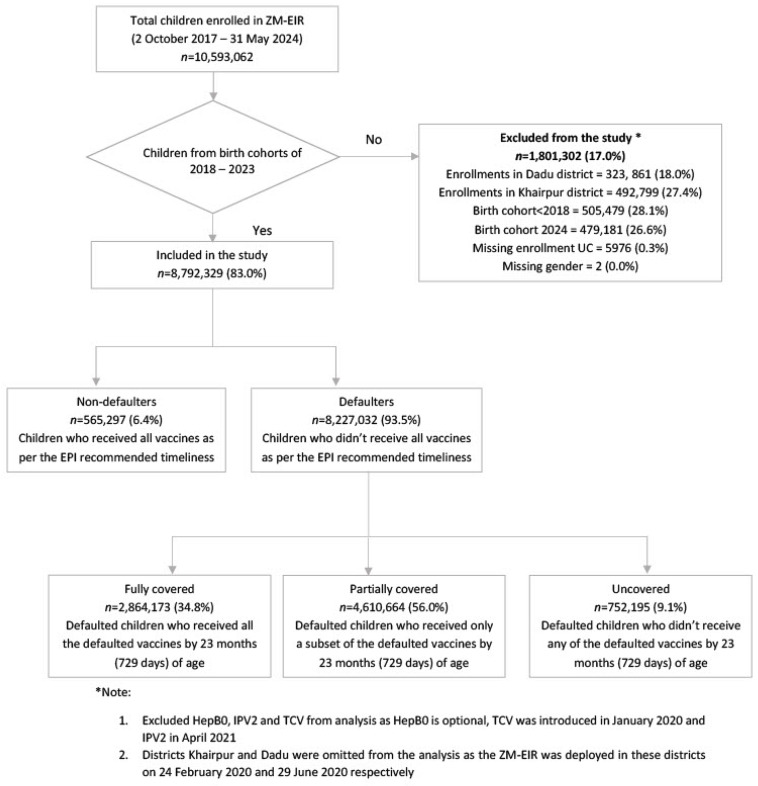
Flowchart representing the inclusion and exclusion criteria of the study population for children enrolled from 2018 to 2023 birth cohorts enrolled in ZM-EIR Sindh, Pakistan.

We used data for 10,593,062 children enrolled in the ZM-EIR between 2 October 2017, and 31 May 2024. Based on specific inclusion criteria, we extracted child-level longitudinal immunization records from the 2018 to 2023 birth cohorts in Sindh, Pakistan. Children from birth cohorts preceding 2018 and from the birth cohort of 2024, and those with missing enrollment UC data and gender were excluded (Figure 1). We also excluded HepB0, IPV-2, and TCV from our analysis, as HepB0 is optional and TCV and IPV-2 were introduced in January 2020 and April 2021. Data from District Khairpur and District Dadu were omitted from the analysis, as the ZM-EIR was deployed in these districts in 2020. This resulted in a 17% reduction of the original dataset of 10.5 million. Our final analytical sample consisted of records for 8,972,329 children. Our analytical period ranges from 1 January 2018 to 31 May 2024.

We extracted data on demographic information (gender, age, birth year, and mother’s education), immunization history (vaccines received, dates of administration, and geo-coordinates of the vaccination sites), and modality of immunization service delivery (fixed, routine outreach, or enhanced outreach). We also gathered details about the place of birth (home, maternity home, or hospital), provision of the caregiver’s CNIC and contact information, whether the caregiver opted for SMS reminder services at the time of the child’s enrollment, and residential areas and subareas (urban vs. rural, non-remote rural vs. remote rural, and urban slums vs. urban non-slums). Missing data were handled differently depending on the type of analysis. For antigen-specific analyses, like assessing antigen-wise coverage and up-to-date coverage, only records with missing information on the vaccination date for that specific antigen were excluded. Only cases where the exact vaccination date was unavailable were excluded to maintain the accuracy of the timeline analysis, even if the child was confirmed to have been vaccinated. This approach preserved the integrity of the analysis while still maintaining an adequate sample size. Similarly, for reporting socio-demographic characteristics and other predictors, missing data were handled on a variable-by-variable basis. If a record had missing information for a particular variable, it was excluded from the analysis for that specific variable. The proportions of missing values for each variable are clearly reported in the footnote of the tables to ensure transparency. For the multivariable analysis, our analytical sample consisted of 16% (1,434,185/8,792,392) of the child-level sample because observations with missing values were excluded because of Stata’s default list-wise deletion.

We followed the Strengthening the Reporting of Observational Studies in Epidemiology (STROBE) guideline to ensure completeness of reporting [32].

### 2.6. Outcome and Measures

The primary outcome of our analysis was the receipt of EPI-recommended vaccinations (BCG, polio, penta, PCV, rotavirus, and measles) for children from the birth cohorts of 2018 to 2023. We included outcome data for children up to 23 months (729 days) of age, with right censoring applied to account for the EPI-recommended target vaccination age for children under two. For the 2022–2023 birth cohorts, adjustments were made to accommodate children who had not yet reached 23 months by the end of the study period. For all birth cohorts, at least a five-month buffer period was provided, as the analysis was conducted until 31 May 2024.

We analyzed immunization coverage in several ways, including antigen-wise coverage, which refers to the proportion of children vaccinated by 23 months in each birth cohort. Cumulative up-to-date coverage was also assessed at key age milestones, namely 6, 12, 18, and 23 months, by dividing the number of children vaccinated at each milestone by the number of children eligible for vaccination at that time. Children who had not yet turned 23 months were excluded from this calculation. Additionally, dropout rates were determined by calculating the percentage difference between the number of children who received the first and last vaccines in the EPI schedule. For instance, the BCG-to-Measles-1 dropout rate was calculated by subtracting the number of children vaccinated with Measles-1 from those vaccinated with BCG and then dividing it by the number of children who received BCG. We report dropout rates across various antigens, including BCG to Penta-1, Penta-2, Penta-3, Measles-1, and Measles-2; Penta-1 to Penta-2, Penta-3, Measles-1, and Measles-2; Penta-2 to Penta-3, Measles-1, and Measles-2; Penta-3 to Measles-1 and Measles-2; and Measles-1 to Measles-2.

Vaccine timeliness was assessed by determining the proportion of children who received each vaccine within the recommended time frame. This analysis was limited to children who received the vaccine and was compared across different birth cohorts. We also compared immunization modalities, such as fixed immunization sites, routine outreach, and enhanced outreach programs, across birth cohorts to better understand coverage patterns.

We categorized children as non-defaulters if they received all vaccines according to the EPI-recommended timeliness range. Children who missed any vaccines, as per the EPI recommended timeliness range (Supplementary Table S1), were classified as defaulters. Children younger than the recommended age for a specific vaccine were not considered defaulters. Among defaulters, we defined those who received all missed vaccines by 23 months of age as fully covered, those who received some missed vaccines as partially covered, and those who received none of the vaccines as not covered (Figure 1). We also conducted an in-depth analysis of defaulter children and compared the sociodemographic characteristics of children based on their defaulter status and examined the impact of external shocks, such as the COVID-19 pandemic and the 2022 floods, on the distribution and coverage of defaulters. For age-appropriate coverage, we considered all children eligible for a specific vaccine and categorized children as uncovered if they did not receive the vaccine they were eligible for, regardless of whether they had reached 23 months of age. Lastly, we used defaulter coverage status as an outcome in a multivariable regression to identify key predictors of defaulters who were not covered for any vaccines.

### 2.7. Statistical Analysis

We reported frequencies and percentages for categorical data and median and interquartile range (IQR) for continuous data, categorized by birth year and defaulter status. The percentage of missing entries for variables such as child’s place of birth, enrollment modality, mother’s education, and SMS reminder enrollment has also been reported in the footnotes of the table. We compared up-to-date coverages at 6, 12, 18, and 23 months across vaccines and by defaulter status across birth cohorts. Univariate and multivariable logistic regression was used to identify predictors of uncovered defaulter children for any missing vaccines. We used a forward stepwise approach for the final multivariable model selection, with gender as a lock term, specifying a *p*-value of 0.05 for entry and 0.10 for removal to identify the model with the lowest Akaike’s Information Criterion (AIC) score [33]. All tests were two-sided, and statistical significance was set at 0.05. Statistical analyses were conducted using Stata, version 17 (StataCorp, College Station, TX, USA). We utilized digital maps in QGIS (version 3.16.7-Hannover) to visualize defaulter coverage by district and birth cohort.

## 3. Results

### 3.1. Overview of Immunization Coverage and Timeliness

**Figure 2:**
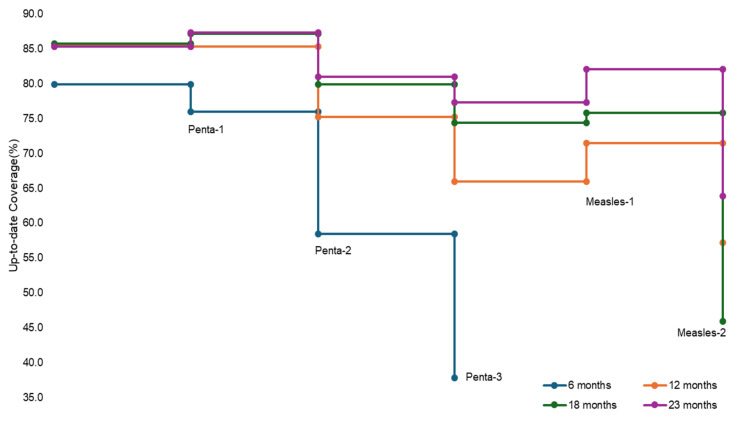
Antigen-wise cumulative up-to-date coverage rates at 6, 12, 18, and 23 months among children from 2018 to 2023 birth cohorts enrolled in ZM-EIR across Sindh, Pakistan, by birth cohort (*n* = 8,792,329)—1 January 2018–31 May 2024.

Between 1 January 2018 and 31 May 2024, 8,792,329 children were enrolled in the ZM-EIR from the birth cohorts of 2018 (11.7%), 2019 (15.4%), 2020 (16.2%), 2021 (16.6%), 2022 (20.3%), and 2023 (19.9%). Overall, across the years, coverage was highest for BCG (2018: 81.8%, 2019: 82.6%, 2020: 87.0%, 2021: 86.8%, 2022: 90.3%, and 2023: 91.2%) and lowest for Measles-2 (2018: 48.2%, 2019: 57.5%, 2020: 65.8%, 2021: 74.4%, 2022: 64.8%) (Table 1). There was a gradual improvement in immunization coverage for all antigens across birth cohorts. Almost 23% more children were fully immunized in 2022 than in the 2018 birth cohort (FIC-M1 without PCV: 47.5% in 2018 to 70.7% in 2022; and FIC-M2 without PCV: 33.5% in 2018 to 55.7% in 2022). Dropout rates declined across the birth cohorts for children enrolled in the EIR (Supplementary Figure S1). Up-to-date coverage at 23 months was higher than the up-to-date coverages at 6, 12, and 18 months for all antigens (Penta-3: 37.8% at 6 months, 66.0% at 12 months, 74.4% at 18 months, and 77.3% at 23 months) (Figure 2). Up-to-date coverage at all ages was the highest for the birth cohort of 2022 (Penta-3: 86.4% at 23 months) and the lowest for the 2018 birth cohort (Penta-3: 64.6% at 23 months) (Supplementary Figure S2).

**Figure 3:**
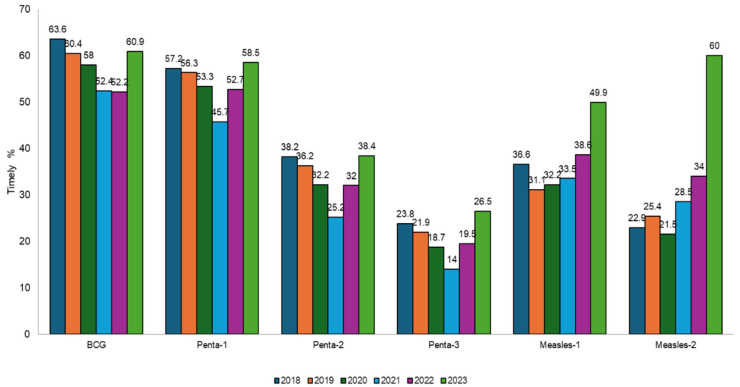
Antigen-wise timeliness among children from 2018 to 2023 birth cohorts enrolled in ZM-EIR across Sindh, Pakistan by birth year.

Overall, vaccine timeliness declined from the 2018 to 2021 birth cohorts but increased from 2022 to 2023 (Figure 3). Amongst all antigens, BCG had the highest percentage of doses administered on time (2018: 63.6%, 2019: 60.4%, 2020: 58%, 2021: 52.4%, 2022: 52.2%, and 2023: 60.9%). The lowest timeliness was observed for Penta-3 (2018: 23.8%, 2019: 21.9%, 2020: 18.7%, 2021: 14.0%, 2022: 19.5%, and 2023: 26.5%) and Measles-2 (2018: 22.9%, 2019: 25.4%, 2020: 21.5%, 2021: 28.5%, 2022: 34%, 2023: 60.0%). Kaplan–Meier curves show that more than 50% of all three doses of the pentavalent vaccine were given after the recommended EPI age for each birth year (Supplementary Figure S3). Median time intervals between doses for all antigens are presented in Supplementary Table S2. These intervals were consistently longer than recommended, with the longest intervals observed for children born in 2021 (Penta-2 to Penta-3: Median = 53 days, IQR = 34–87; recommended interval = 28 days).

**Figure 4:**
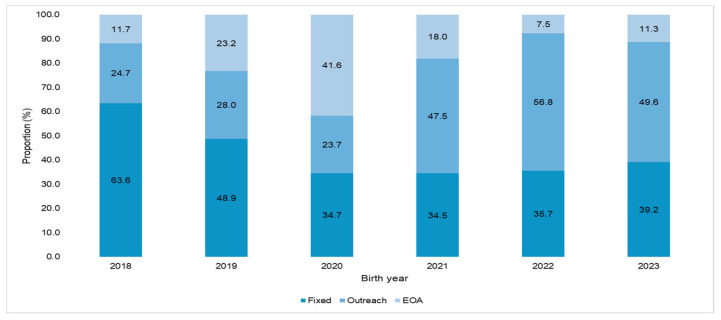
Comparison of immunization doses administered through different modalities among children from 2018 to 2023 birth cohorts enrolled in ZM-EIR across Sindh, Pakistan (*n* = 8,792,329).

Examining the pattern of immunization modalities through which children were vaccinated, we observe that, for children born in 2018 and 2019, the majority of vaccinations were given through fixed immunization sites (63.6% and 48.9%, respectively). However, for children born in 2020, most of the vaccinations (41.6%) were provided through enhanced outreach activities (Figure 4). For later birth cohorts from 2021 to 2023, almost half of the immunization doses were administered through outreach activities (2021: 47.5%, 2022: 56.8%, and 2023: 49.6%). Antigen-wise comparison of immunization doses administered through fixed, outreach activities, and EOA by birth year and vaccines are presented in Supplementary Figure S4. Across all birth cohorts, vaccines given earlier in life (BCG) were mainly given at fixed sites (Range: 46.0–76.4%), while for later vaccines such as Penta-3, Measles-1, and Measles-2, a higher proportion were given through outreach (range: Penta-3 = 24.9–59.9%, Measles-1 = 23.4–65.8%, and Measles-2 = 20.7–65.8%) or EOA activities (range: Penta-3 = 7.8–44.8%, Measles-1 = 10.4–43.9%, and Measles-2 = 2.4–53.5%).

### 3.2. Trends in Coverage and Predictors of Immunization Defaulters

**Figure 5:**
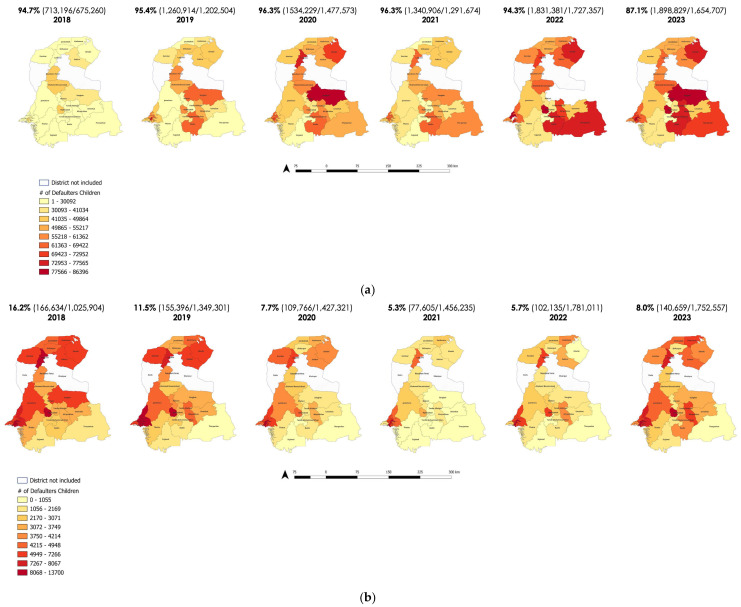
(**a**) Number of defaulter children and (**b**) covered and uncovered defaulter children based on enrollment district from 2018 to 2023 birth cohorts enrolled in ZM-EIR Sindh, Pakistan by birth year.

Among all children in our sample, 6.4% (565,297/8,792,329) were non-defaulters, and 93.6% (8,227,032/8,792,329) were defaulters (Table 2). Of these defaulters, 34.8% (2,864,173/8,227,032) were fully covered, 56.0% (4,610,664/8,227,032) were partially covered, and 9.1% (752,195/8,227,032) were not covered for any of the recommended vaccines by 23 months of age. The proportion of defaulters decreased from 94.7% to 87.1%, while the proportion of uncovered defaulters declined from 16.2% to 8.0% across the birth cohorts from 2018 to 2023. The proportion of defaulters (94.7% vs. 87.1%) and uncovered defaulters (16.2% vs. 8.0%) decreased across the birth cohorts from 2018 to 2023 (Figure 5). The birth cohorts of 2020 and 2021 saw the highest default rates (96.2% and 96.5%), respectively.

By examining the socio-demographic characteristics of the different defaulter categories, we observe that there was no difference in the proportion of male and female children who were non-defaulters (male: 6.4% vs. female: 6.5%) or defaulters (male: 93.6% vs. female: 93.5%) (Table 2). In terms of geographic distribution, a higher proportion of defaulters was observed in remote rural areas (98%), followed by non-remote rural areas (96.5%) and urban areas (91.2%). However, urban areas had more uncovered defaulters (12.7%), compared to both remote rural areas (3%) and non-remote rural areas (5%). Urban non-slum areas had a higher proportion of defaulters than urban slums (96.7% vs. 91.2%), but more defaulters in urban slums (12.7%) were uncovered. Children born at home had a higher default rate (96.4%) than those born in maternity homes (92.2%) and hospitals (88.9%). A greater proportion of these hospital-born children, however, remained uncovered (14.1%) compared to those born at home (7.4%). Children enrolled through EOAs had a higher default rate (98.1%) compared to those enrolled through fixed sites (90.9%), outreach (95.8%), and mobile immunization vans (96.9%). Yet, a higher proportion of defaulters remained uncovered at fixed sites (13.8%). Among children whose mothers had no education, 97.1% defaulted compared to the lower default rates among those with higher education levels (6–8 years: 93.3%, 9–10 years: 89.7%, and >11 years: 86.3%). Conversely, 23.5% of defaulter children with highly educated mothers (>11 years of education) were not covered for any vaccine, compared to only 8.7% of defaulter children with mothers who had no education.

Up-to-date coverage rates at 6, 12, 18, and 23 months were higher for non-defaulters compared to defaulters (Table 3). The difference in coverage rates was more pronounced for vaccines administered later in the schedule (difference for up-to-date coverage at 6 months between non-defaulters and defaulters; BCG: 18.3%; Penta-1: 20%; Penta-2: 36.5%; and Penta-3: 56.5%). However, as children grew older, the difference between non-defaulters and defaulters became less (BCG: 18.3% at 6 months vs. 10.2% at 23 months). Nevertheless, the difference remained more pronounced for later vaccines (Measles-1: 40.4% at 12 months vs. 18.6% at 23 months). The difference between age-appropriate immunization coverage of non-defaulters and defaulters decreased across birth cohorts (FIC-M1 (without PCV) at 23 months: 34.5% in 2018 vs. 23.3% in 2020) (Supplementary Tables S3.1–S3.4).

By overlaying the proportion of defaulter children with key external shocks between 2018 and 2023, we saw that, even prior to the COVID-19-induced disruptions, (1 January 2018 to 22 March 2020), the percentage of defaulters was high, at 95.7% (Table 4). The proportion of defaulters remained high (>95%) throughout the COVID-19 lockdowns and disruptions, as well as the 2022 floods, only decreasing gradually at the start of 2023 (to 82.4%). The proportion of uncovered defaulters was the highest during the pre-pandemic time (1 January 2018–22 March 2020) and during the initial lockdowns (23 March–9 May 2020) (>10%) but then reduced to almost half during the pandemic’s last duration (17.1% vs. 6.6%) (Table 4; Figure 5).

Table 5 reports the predictors of uncovered defaulters vs. immunized children among children from the 2018 to 2023 birth cohorts. Children born in maternity homes (OR = 0.81, 95% CI: 0.79–0.83, *p* < 0.001) and at home (OR = 0.83, 95% CI: 0.82–0.84, *p* < 0.001) were less likely to be uncovered defaulters compared to those born in hospitals. Those born in remote rural areas demonstrated a decreased likelihood of being uncovered defaulters (OR = 0.80, 95% CI: 0.78–0.83, *p* < 0.001), while those in urban areas (OR = 1.54, 95% CI: 1.52–1.56, *p* < 0.001) were more likely to be uncovered defaulters compared to those in non-remote rural areas. Enrollment modality also influenced the default rates. Children enrolled through fixed sites (OR = 2.11, 95% CI: 2.08–2.15, *p* < 0.001) and mobile immunization vans (OR = 1.41, 95% CI: 1.13–1.77, *p* = 0.003) had a higher risk of being uncovered, while those enrolled through the EOAs (OR = 0.69, 95% CI: 0.67–0.72, *p* < 0.001) were less likely to be uncovered defaulters compared to those enrolled through outreach. Children of mothers with lower education levels were less likely to be uncovered defaulters compared to those with more education (0 years: OR = 0.72, 95% CI: 0.71–0.74, *p* < 0.001; 9–10 years: OR = 0.78, 95% CI: 0.76–0.81, *p* < 0.001). Later birth cohorts showed lower odds of being uncovered defaulters compared to those born in 2018 and 2019 (OR = 0.57, 95% CI: 0.57–0.58, *p* < 0.001), 2020 (OR = 0.40, 95% CI: 0.39–0.41, *p* < 0.001), 2021 (OR = 0.25, 95% CI: 0.24–0.25, *p* < 0.001), 2022 (OR = 0.25, 95% CI: 0.24–0.25, *p* < 0.001), and 2023 (OR = 0.34, 95% CI: 0.33–0.34, *p* < 0.001).

## 4. Discussion

Our analysis of routine immunization coverage for children enrolled in the ZM-EIR from the 2018 to 2023 birth cohorts in Sindh, Pakistan demonstrates an over 20% increase in full immunization coverage, accompanied by a decline in dropouts over the 5-year period. Fewer children received vaccines timely from the 2018 to 2021 birth cohorts. However, there was some improvement from 2022 to 2023. There was a high proportion of defaulters (>90%) in all birth cohorts, particularly in the most marginalized groups (remote rural areas, children born at home, those enrolled in outreach, EOAs or MIVs, and those with uneducated mothers). The proportion of uncovered defaulters peaked before and during early COVID-19 lockdowns but was significantly reduced towards the end of the pandemic. Factors such as residence, place of birth, enrollment modality, maternal education, and birth year influenced a child’s likelihood of being an uncovered defaulter.

We observed a consistent upward trend in immunization coverage rates over the 5-year period from 2018 to 2023. However, this overall trend masks the underlying inequities and challenges that hinder efforts to achieve universal immunization targets. Recent disruptions associated with the pandemic, and the severe flooding of 2022, have been reported to reverse some of the positive immunization trends and led to reduced coverage rates in Pakistan [15,17]. The findings from our earlier work also show that one in two children missed routine vaccinations during the first lockdown in Sindh [20]. While we observed an overall increasing trend in coverage, even in the COVID-19 and post-COVID-19 years, our findings do provide evidence of disruptions during the peak pandemic years (2020 and 2021), where we observed a high default rate (>95%), and a decline in vaccine timeliness. Despite these challenges, the overall improvement in coverage can be attributed to the fact that nearly half of all vaccine doses administered during these peak pandemic years (2020 and 2021) were delivered through outreach efforts or EOAs, which could have likely mitigated the negative impact of the pandemic by focusing on catching up on missed vaccines [34]. A 2023 UNICEF report also identified Pakistan as one of five South Asian countries that successfully restored immunization rates to pre-pandemic levels through initiatives targeting high-risk and vulnerable communities [35]. In Sindh particularly, policies including a dedicated workforce for EPI tasks, adaptive outreach strategies, flexible vaccination sites, strengthened supply chains, and enhanced public awareness helped sustain and recover routine immunization [36].

The timeliness of vaccination also showed varying trends. Fewer children received their vaccines on time from the 2018 to 2021 birth cohorts, which could partly be explained by disruptions caused by the COVID-19 pandemic. These disruptions affected the regular vaccination schedule, leading to delays and missed doses during the pandemic years. However, some improvements in timeliness were observed from the 2022 and 2023 birth cohorts, signaling a recovery as routine health services resumed, and catch-up campaigns were initiated. Timeliness was particularly low for vaccines administered later in life, such as Penta-3, Measles-1, and Measles-2, which are given at 14 weeks, 9 months, and 15 months of age, respectively, illustrating challenges to maintaining continuity in the vaccination schedule. Our findings are in line with other studies in Pakistan that report lower timeliness for older children because of mainly logistic challenges, which result in lower compliance with vaccination appointments [37,38]. Our analysis of age-specific vaccines and timeliness fills an important gap in the literature by providing detailed insights into how delays and dropouts occur at different stages of the immunization schedule. This information is crucial for identifying points in the schedule where targeted interventions can improve coverage and timeliness.

Consistent with global statistics, we found that BCG coverage was consistently the highest across all birth cohorts, whereas Penta-3 and Measles-2 coverage remained the lowest [37,39,40,41]. Additionally, while coverage rates were higher among non-defaulters compared to defaulters throughout, this difference was more pronounced for vaccines administered later in life. BCG often achieves higher coverage due to its administration at birth or shortly thereafter (especially in health facilities), whereas later vaccines, such as Penta-3 and Measles-2, face hurdles such as logistical challenges, low awareness, and vaccine hesitancy [18,42]. Lower coverage for Penta-3 and Measles-2 may also reflect gaps in the continuity of vaccination campaigns, especially in remote and underserved areas where health system disruptions, including reduced outreach services during the pandemic, were most pronounced [43]. Our results also confirm that earlier vaccines like BCG were primarily administered at fixed sites, whereas later vaccines were often delivered through supplementary and catch-up activities (outreach, EOAs, etc.). While this reliance on supplementary immunization strategies ensured coverage for many children, it highlights the underlying gaps in the regular immunization delivery system. There is a need for in-depth studies to understand the factors contributing to the decline in vaccination coverage during the critical 9–15-month period for designing and implementing tailored strategies to address these gaps.

We found that children from remote rural areas, those born at home, those enrolled in the registry through modalities other than fixed sites, and those with uneducated mothers had higher default rates. For children born at home, the higher defaulter rates may stem from limited postnatal care access and cultural practices that reduce formal healthcare visits soon after birth, hindering early integration into the immunization system [44]. Despite more children defaulting in these groups, they had a lower risk of being left uncovered for missed vaccines up to 23 months of age. These findings are somewhat counterintuitive and contrast with the previous research that indicates that the factors associated with parental education, place of birth, and residence typically have a protective effect against missing vaccinations [45,46]. Our contradictory findings could possibly be because, while the most marginalized groups are more likely to default on vaccines, catch-up immunization strategies have been successful in reaching these high-risk populations who may otherwise face barriers to accessing vaccination services [34,42]. Our data also suggest that reliance on supplementary immunization activities (especially for later vaccines and during 2021–2023) could have helped maintain coverage rates, ensuring broader reach among traditionally marginalized groups and addressing the needs of these high-risk populations. It is important to note that, while much of the literature focuses on the factors associated with the risk of defaulting on vaccines, our novel approach—assessing predictors of uncovered defaulters—provides a fresh perspective on the likelihood of achieving coverage among defaulters for missed immunizations.

While our study provides valuable insights into immunization coverage in Sindh province, Pakistan, it has certain limitations. For the multivariable analysis, our analytical sample consisted of only 16% (1,434,185/8,792,392) of the original sample, affecting the reliability and robustness of our findings. As such, the multivariable variables results should be interpreted carefully, particularly in terms of the generalizability of our findings to other settings. Our focus was to provide preliminary insight to the readers on the direction of our results rather than the precise magnitude of the effect size. We emphasize the need for further research with larger sample sizes and combining quantitative data with qualitative data to provide additional insights and help contextualize our findings. It is also important to interpret the lower coverage rates observed in the 2022 and 2023 birth cohort with caution, as many children born in that year may not yet be due for several vaccines scheduled later in life, such as Penta-3 and Measles-2. To avoid the impact of this unequal time of analyses (less than 2 years) for children from the last two birth cohorts (2022 and 2023) and to ensure the reliability of our interpretations, we used proportions for outcomes such as timeliness of vaccines. Some of the vaccination data were collected retrospectively, which may have led to missing information, particularly regarding vaccination dates, due to caregivers’ inability to recall exact dates. While our study identifies associations between factors and the likelihood of being an uncovered defaulter, it is indeed descriptive in nature and does not aim to establish causality. However, as a descriptive analysis, the findings highlight trends and potential relationships, which should be further explored through more robust study designs. Additionally, despite in-built checks in the ZM-EIR app to ensure data reliability, such as locking inaccurate entries and restricting field formats, variability in data entry practices may still occur. Lastly, the generalizability of our results may be limited, as the data are specific to Sindh province and may not fully represent immunization trends in other regions of Pakistan or countries with different health systems. Following the decentralization of the EPI, the provinces have implemented immunization policies according to their contextual requirements. However, the large overarching direction of mass disease prevention policies and agenda remain unified for the country. As such, our findings provide a microcosmic view of Pakistan’s larger immunization landscape, as Sindh’s efforts, policies, and challenges are reflective of nationwide trends. Moreover, challenges and contexts faced by immunization systems are common across low-resource settings, and this commonality suggests that our findings will provide valuable insights for other regions facing similar challenges.

Our findings carry important implications for the immunization efforts in Sindh, Pakistan, and similar low-resource settings. We leveraged big data derived from records of >8 million children to provide a comprehensive and in-depth analysis of immunization coverage, timeliness, and defaulters in Sindh, Pakistan. Our analysis goes beyond merely assessing overall coverage and timeliness; it provides a comprehensive examination of defaulter children, their sociodemographic characteristics, and the risk factors associated with missed vaccines, while also exploring coverage rates in the context of external shocks. The successful recovery of immunization rates, despite major disruptions such as the COVID-19 pandemic and the 2022 floods, reflects the resiliency of the system and the effectiveness of timely, targeted data-driven interventions and adaptive strategies aimed at reaching marginalized groups. This also emphasizes the need for continued investment in vaccination infrastructure, especially in remote and high-risk regions. To improve coverage and address disparities, a coordinated effort among all stakeholders (policymakers, provincial and national governments, health departments, international and local multilateral organizations, etc.) is required to enhance outreach and community engagement, particularly in high-default areas. Integrating real-time data into vaccination programs can further facilitate evidence-based planning and implementation.

Future strategies should focus on strengthening vaccine delivery systems to ensure equitable access, address systemic barriers, and improve timeliness, especially for vaccines scheduled beyond infancy. Additionally, systematic follow-up with defaulters is crucial to completing vaccination schedules. Further work is needed to investigate the underlying reasons for vaccine-specific dropout rates, evaluate the effectiveness of outreach and catch-up vaccination campaigns to refine strategies that effectively target high-risk populations, and assess the long-term impact of external disruptions like COVID-19 and flooding. Future studies with experimental designs are also needed to establish causal relationships between various factors and the likelihood of being uncovered defaulters.

## 5. Conclusions

Our study demonstrates improvements in immunization coverage across birth cohorts in Sindh, Pakistan, alongside a reduction in dropout rates from 2018 to 2023. While the timeliness of vaccination declined from 2018 to 2021, it showed recovery in 2022 and 2023. Despite a high proportion of children defaulting on due vaccines, the majority were covered for missed vaccinations, while the percentage of uncovered defaulters decreased over time. Key predictors of uncovered defaulters include birthplace, residential area, enrollment modality, and maternal education. However, it is important to acknowledge that these findings are based on the available data, which may not be fully representative of all populations. Moving forward, a collaborative approach involving all stakeholders is essential to address systemic barriers and improve outreach efforts. To create a more robust immunization system, the focus should be on addressing improving vaccine accessibility, particularly in underserved areas, and implementing targeted follow-up and catch-up vaccination strategies. Strengthening data systems for real-time monitoring, increasing public awareness, and engaging communities, especially through maternal education, are essential to reduce default rates. Future research should focus on understanding the factors contributing to high default rates and identifying reasons for vaccine dropouts to guide the development of targeted strategies and policies to ensure comprehensive coverage for defaulters. Further studies with experimental designs are needed to establish the causal relationships between various factors and the likelihood of being uncovered as defaulters. Building a robust and adaptable immunization system is vital for safeguarding the health of all children in Pakistan and ensuring that they receive the vaccines they need for a healthier future.

## Figures and Tables

**Table 1:** Antigen-wise coverage ^a^ among children from 2018 to 2023 birth cohorts enrolled in ZM-EIR across Sindh, Pakistan (*n* = 8,792,329)—1 January 2018–31 May 2024.

	2018 (*n* = 1,025,904)		2019 (*n* = 1,349,301)		2020 (*n* = 1,427,321)		2021 (*n* = 1,456,235)		2022 (*n* = 1,781,011)		2023 ^b^ (*n* = 1,752,557)	
	*n*	%	*n*	%	*n*	%	*n*	%	*n*	%	*n*	%
BCG	838,759	81.8	1,115,125	82.6	1,241,736	87.0	1,264,245	86.8	1,608,041	90.3	1,598,400	91.2
Penta-1	795,965	77.6	1,091,995	80.9	1,230,126	86.2	1,274,994	87.6	1,626,996	91.4	1,555,632	88.8
OPV-1	793,680	77.4	1,090,271	80.8	1,230,836	86.2	1,275,553	87.6	1,627,170	91.4	1,555,720	88.8
PCV-1	795,716	77.6	1,091,409	80.9	1,229,525	86.1	1,274,610	87.5	1,626,875	91.4	1,555,427	88.8
Rota-1	726,949	70.9	1,071,660	79.4	1,212,780	85.0	1,262,421	86.7	1,619,480	90.9	1,554,627	88.7
Penta-2	707,046	68.9	1,005,418	74.5	1,151,892	80.7	1,211,603	83.2	1,519,045	85.3	1,352,710	77.2
OPV-2	706,414	68.9	1,004,673	74.5	1,152,511	80.8	1,211,894	83.2	1,518,881	85.3	1,352,609	77.2
PCV-2	707,029	68.9	1,005,008	74.5	1,151,566	80.7	1,211,381	83.2	1,518,937	85.3	1,352,683	77.2
Rota-2	631,306	61.5	971,064	72.0	1,120,812	78.5	1,189,101	81.7	1,507,849	84.7	1,351,851	77.1
Penta-3	662,556	64.6	962,962	71.4	1,143,243	80.1	1,234,864	84.8	1,521,773	85.4	1,219,269	69.6
OPV-3	662,497	64.6	963,027	71.4	1,143,648	80.1	1,234,633	84.8	1,521,354	85.4	1,219,021	69.6
PCV-3	662,883	64.6	962,774	71.4	1,142,820	80.1	1,234,380	84.8	1,521,468	85.4	1,219,208	69.6
IPV-1	701,996	68.4	1,001,399	74.2	1,179,686	82.7	1,269,917	87.2	1,565,805	87.9	1,287,079	73.4
Measles-1	679,936	66.3	972,423	72.1	1,163,588	81.5	1,264,048	86.8	1,505,384	84.5	744,064	42.5
Measles-2	494,924	48.2	775,845	57.5	938,988	65.8	1,083,596	74.4	1,154,170	64.8	85,543	4.9
FIC-M1 (without PCV)	486,961	47.5	710,973	52.7	907,318	63.6	988,424	67.9	1,258,668	70.7	622,340	35.5
FIC-M1 (with PCV)	486,651	47.4	710,110	52.6	906,145	63.5	987,617	67.8	1,257,876	70.6	622,050	35.5
FIC-M1 (with Rota)	436,596	42.6	687,068	50.9	881,595	61.8	968,413	66.5	1,247,209	70.0	620,977	35.4
FIC-M2 (without PCV)	344,085	33.5	561,749	41.6	740,764	51.9	860,908	59.1	992,417	55.7	77,370	4.4
FIC-M2 (with PCV)	343,927	33.5	561,164	41.6	739,867	51.8	860,253	59.1	991,914	55.7	77,348	4.4
FIC-M2 (with Rota)	308,873	30.1	543,478	40.3	720,467	50.5	844,300	58.0	984,348	55.3	77,146	4.4

^a^ Coverage: Children who received a particular vaccine within 729 days; FIC-M1: (1) Without PCV = BCG + OPV1-3 + Penta1-3 + M1, (2) With PCV = BCG + OPV1-3 + Penta1-3 + PCV1-3 + M1 (3), With Rota = BCG + OPV1-3 + Penta1-3 + PCV1-3 + Rota1-2 + M1; FIC-M2: (1) Without PCV = BCG + OPV1-3 + Penta1-3 + M1-2 (2) With PCV = BCG + OPV1-3 + Penta1-3 + PCV1-3 + M1-2, (3) With Rota = BCG + OPV1-3 + Penta1-3 + PCV1-3 + Rota1-2 + M1-2; ^b^ Lower coverage rates in 2023 birth cohort, as children might still not be due for these vaccines.

**Table 2:** Socio-demographic characteristics of children enrolled in ZM-EIR by defaulter status (*n* = 8,792,329)—1 January 2018–31 May 2024.

	Non-Defaulters		Defaulters		Defaulters						Total	
					Fully Covered		Partially Covered		Uncovered			
	*n*	%	*n*	%	*n*	%	*n*	%	*n*	%	*n*	%
**Total**	** 565,297 **	**6.4**	**8,227,032**	**93.6**	** 2,864,173 **	**34.8**	** 4,610,664 **	**56.0**	** 752,195 **	**9.1**	** 8,792,329 **	**100.0**
**Sex**												
Male	293,744	6.4	4,302,970	93.6	1,497,302	34.8	2,410,968	56.0	394,700	9.2	4,596,714	100.0
Female	271,553	6.5	3,924,062	93.5	1,366,871	34.8	2,199,696	56.1	357,495	9.1	4,195,615	100.0
**Residential Area**												
Remote Rural	8871	2.0	442,770	98.0	143,483	32.4	285,962	64.6	13,325	3.0	451,641	100.0
Non-remote Rural	115,976	3.5	3,230,403	96.5	1,212,758	37.5	1,857,466	57.5	160,179	5.0	3,346,379	100.0
Urban	440,450	8.8	4,553,859	91.2	1,507,932	33.1	2,467,236	54.2	578,691	12.7	4,994,309	100.0
**Residential sub-area**												
Urban non-slum	124,847	3.3	3,673,173	96.7	1,356,241	36.9	2,143,428	58.4	173,504	4.7	3,798,020	100.0
Urban slum	440,450	8.8	4,553,859	91.2	1,507,932	33.1	2,467,236	54.2	578,691	12.7	4,994,309	100.0
**Place of birth ^a^**												
Hospital	142,040	11.1	1,134,712	88.9	414,501	36.5	560,463	49.4	159,748	14.1	1,276,752	100.0
Maternity Home	11,875	7.8	141,012	92.2	50,402	35.7	76,329	54.1	14,281	10.1	152,887	100.0
Home	25,053	3.6	666,940	96.4	224,563	33.7	392,954	58.9	49,423	7.4	691,993	100.0
**Enrollment Event ^b^**												
Fixed Site	420,099	9.1	4,186,423	90.9	1,561,953	37.3	2,046,380	48.9	578,090	13.8	4,606,522	100.0
Outreach	117,102	4.2	2,645,010	95.8	882,113	33.4	1,643,140	62.1	119,757	4.5	2,762,112	100.0
Enhanced outreach activities	25,716	1.9	1,321,517	98.1	407,552	30.8	863,216	65.3	50,749	3.8	1,347,233	100.0
Mobile Immunization Vans	2380	3.1	74,080	96.9	12,553	16.9	57,928	78.2	3599	4.9	76,460	100.0
** Mother’s Education ^c^ (years)**												
0	17,964	2.9	591,010	97.1	202,282	34.2	337,458	57.1	51,270	8.7	608,974	100.0
1–5	29,659	4.7	600,493	95.3	229,640	38.2	308,233	51.3	62,620	10.4	630,152	100.0
6–8	6006	6.7	83,604	93.3	29,936	35.8	41,759	49.9	11,909	14.2	89,610	100.0
9–10	8546	10.3	74,757	89.7	25,145	33.6	35,087	46.9	14,525	19.4	83,303	100.0
≥11	8797	13.7	55,370	86.3	18,961	34.2	23,390	42.2	13,019	23.5	64,167	100.0
**Birth year**												
2018	44,430	4.3	981,474	95.7	232,144	23.7	582,696	59.4	166,634	17.0	1,025,904	100.0
2019	56,217	4.2	1,293,084	95.8	379,744	29.4	757,944	58.6	155,396	12.0	1,349,301	100.0
2020	54,663	3.8	1,372,658	96.2	492,752	35.9	770,140	56.1	109,766	8.0	1,427,321	100.0
2021	50,869	3.5	1,405,366	96.5	505,144	35.9	822,617	58.5	77,605	5.5	1,456,235	100.0
2022	103,792	5.8	1,677,219	94.2	643,981	38.4	931,103	55.5	102,135	6.1	1,781,011	100.0
2023	255,326	14.6	1,497,231	85.4	610,408	40.8	746,164	49.8	140,659	9.4	1,752,557	100.0
** Enrollment Age (months)**												
0–1	532,968	10.9	4,362,788	89.1	2,594,212	59.5	1,235,934	28.3	532,642	12.2	4,895,756	100.0
2–3	19,603	1.0	1,887,342	99.0	138,447	7.3	1,620,037	85.8	128,858	6.8	1,906,945	100.0
4–6	3075	0.4	841,770	99.6	55,853	6.6	766,891	91.1	19,026	2.3	844,845	100.0
7–9	3046	0.7	438,778	99.3	24,174	5.5	394,109	89.8	20,495	4.7	441,824	100.0
10–12	909	0.3	340,091	99.7	20,387	6.0	313,312	92.1	6392	1.9	341,000	100.0
13–15	4440	3.3	128,620	96.7	8445	6.6	97,502	75.8	22,673	17.6	133,060	100.0
16–18	1242	1.0	126,459	99.0	14,942	11.8	106,817	84.5	4700	3.7	127,701	100.0
19–21	5	0.0	51,786	100.0	5499	10.6	46,248	89.3	39	0.1	51,791	100.0
≥22	9	0.0	49,398	100.0	2214	4.5	29,814	60.4	17,370	35.2	49,407	100.0
**Age at Vaccination (in months)**	**Median**	**IQR ^e^**	**Median**	**IQR ^e^**	**Median**	**IQR ^e^**	**Median**	**IQR ^e^**	**Median**	**IQR ^e^**	**Median**	**IQR ^e^**
BCG/OPV-0	0.23	0.10–0.43	0.72	0.23–2.04	0.43	0.20–0.89	1.94	0.53–3.98	0.16	0.03–0.39	0.66	0.23–2.00
Penta-1/OPV-1/PCV-1	1.51	1.45–1.64	2.27	1.71–3.81	2.04	1.61–2.83	2.79	1.91–5.06	1.58	1.48–1.78	2.14	1.64–3.58
Penta-2/OPV-2/PCV-2	2.60	2.47–2.76	4.21	3.09–6.64	3.75	2.99–5.26	4.96	3.45–8.18	2.63	2.50–2.83	4.01	2.96–6.34
Penta-3/OPV-3/PCV-3	3.68	3.48–3.88	6.51	4.67–9.99	5.82	4.54–8.32	7.56	5.16–11.57	3.72	3.55–3.91	6.15	4.37–9.60
Measles-1	9.14	9.01–9.34	10.42	9.44–12.59	10.16	9.34–11.64	10.91	9.66–13.77	9.17	9.04–9.40	10.26	9.34–12.36
Measles-2	15.15	15.02–15.39	16.60	15.48–18.44	16.50	15.45–18.21	16.83	15.71–18.84	15.12	15.02–15.38	16.40	15.35–18.21
	***n***	**%**	***n***	**%**	***n***	**%**	***n***	**%**	***n***	**%**	***n***	**%**
** Provision of Contact Number**												
Provided	380,714	12.0	2,792,813	88.0	1,042,478	37.3	1,399,011	50.1	351,324	12.6	3,173,527	100.0
Not provided	184,583	3.3	5,434,219	96.7	1,821,695	33.5	3,211,653	59.1	400,871	7.4	5,618,802	100.0
** Provision of CNIC Numbers**												
Provided	109,662	14.8	629,818	85.2	250,918	39.8	283,780	45.1	95,120	15.1	739,480	100.0
Not provided	455,635	5.7	7,597,214	94.3	2,613,255	34.4	4,326,884	57.0	657,075	8.6	8,052,849	100.0
**SMS Reminders ^d^**												
Opted	195,718	3.4	5,546,850	96.6	1,888,981	34.1	3,250,315	58.6	407,554	7.3	5,742,568	100.0
Not opted	363,425	12.6	2,518,556	87.4	953,553	37.9	1,259,358	50.0	305,645	12.1	2,881,981	100.0

^a^ 75.9% of observations for place of birth are missing in total, 68.4% of observations were missing for non-defaulters, 75.9% of observations missing for fully covered, 77.5% of observations missing for partially covered, and 70.2% for the not covered cohort. 0.05% refused to answer for all the cohorts. ^b^ 2 observations for enrollment modality are missing in total, and also, 2 observations for fully covered cohort are missing. ^c^ 83.2% of observations for mother’s education are missing in total, 87.4% of observations were missing for non-defaulters, 82.3% of observations missing for fully covered, 83.8% of observations missing for partially covered, and 79.5% for the not covered cohort. 0.04% refused to answer for all the cohorts. ^d^ 65.3% of observations for approved reminders are missing in total, 34.6% of observations were missing for non-defaulters, 66.0% of observations missing for fully covered, 70.5% of observations missing for partially covered, and 54.2% for the not covered cohort. ^e^ Interquartile Range (25–75%). Abbreviations: BCG, Bacille Calmette-Guérin; OPV, oral polio vaccine; Penta, pentavalent vaccine, including vaccines against diphtheria, tetanus, pertussis, hepatitis B, and hemophilus influenza; PCV, pneumococcal conjugate vaccine. Non-defaulter: children who received all the vaccines as per EPI recommended timeliness. Fully covered defaulters: children who received all the defaulted vaccines by 23 months of age. Partially covered defaulters: Children who received a few of the defaulted vaccines by 23 months of age. Not covered defaulters: Children who did not receive any of the defaulted vaccines by 23 months of age.

**Table 3:** Antigen-wise up-to-date coverage rates at 6, 12, 18, and 23 months among children from 2018 to 2023 birth cohorts enrolled in ZM-EIR across Sindh, Pakistan by defaulter status (*n* = 8,792,329)—1 January 2018–31 May 2024.

	6 Months					12 Months					18 Months					23 Months				
	Non-Defaulter (*n* = 524,646)		Defaulter (*n* = 8,136.916)		% Difference	Non-Defaulter (*n* = 370,589)		Defaulter (*n* = 7,359,264)		% Difference	Non-Defaulter (*n* = 298,756)		Defaulter (*n* = 6,587,052)		% Difference	Non-Defaulter (*n* = 239,457)		Defaulter (*n* = 5,713,459)		% Difference
	*n*	%	*n*	%	%	*n*	%	*n*	%	%	*n*	%	*n*	%	%	*n*	%	*n*	%	%
BCG	509,465	97.1	6,413,184	78.8	18.3	357,525	96.5	6,246,032	84.9	11.6	286,365	95.9	5,621,463	85.3	10.5	227,772	95.1	4,850,521	84.9	10.2
Penta-1	496,998	94.7	6,083,630	74.8	20.0	358,238	96.7	6,238,134	84.8	11.9	286,964	96.1	5,717,622	86.8	9.3	228,181	95.3	4,973,359	87.0	8.2
OPV-1	496,914	94.7	6,083,289	74.8	20.0	358,224	96.7	6,238,586	84.8	11.9	286,961	96.1	5,717,445	86.8	9.3	228,177	95.3	4,972,580	87.0	8.3
PCV-1	497,005	94.7	6,082,373	74.8	20.0	358,241	96.7	6,236,830	84.7	11.9	286,966	96.1	5,716,358	86.8	9.3	228,182	95.3	4,972,040	87.0	8.3
Rota-1	496,778	94.7	6,052,002	74.4	20.3	357,790	96.5	6,177,910	83.9	12.6	286,524	95.9	5,645,399	85.7	10.2	227,757	95.1	4,895,953	85.7	9.4
Penta-2	486,546	92.7	4,573,545	56.2	36.5	356,106	96.1	5,461,257	74.2	21.9	284,963	95.4	5,215,593	79.2	16.2	226,318	94.5	4,595,966	80.4	14.1
OPV-2	486,467	92.7	4,573,404	56.2	36.5	356,109	96.1	5,461,734	74.2	21.9	284,971	95.4	5,216,056	79.2	16.2	226,331	94.5	4,596,043	80.4	14.1
PCV-2	486,560	92.7	4,573,617	56.2	36.5	356,115	96.1	5,461,147	74.2	21.9	284,970	95.4	5,215,220	79.2	16.2	226,323	94.5	4,595,460	80.4	14.1
Rota-2	478,787	91.3	4,364,763	53.6	37.6	350,337	94.5	5,156,110	70.1	24.5	280,085	93.8	4,888,073	74.2	19.5	222,177	92.8	4,277,523	74.9	17.9
Penta-3	476,787	90.9	2,798,437	34.4	56.5	353,051	95.3	4,749,004	64.5	30.7	282,117	94.4	4,840,118	73.5	21.0	223,696	93.4	4,379,560	76.7	16.8
OPV-3	476,572	90.8	2,796,202	34.4	56.5	352,965	95.2	4,748,636	64.5	30.7	282,097	94.4	4,840,010	73.5	20.9	223,699	93.4	4,379,322	76.6	16.8
PCV-3	476,789	90.9	2,797,519	34.4	56.5	353,053	95.3	4,748,864	64.5	30.7	282,118	94.4	4,839,417	73.5	21.0	223,703	93.4	4,378,584	76.6	16.8
IPV-1	476,449	90.8	3,222,400	39.6	51.2	356,352	96.2	5,165,548	70.2	26.0	285,615	95.6	5,171,670	78.5	17.1	226,618	94.6	4,652,844	81.4	13.2
Measles-1			-	-	-	354,290	95.6	4,063,989	55.2	40.4	298,691	100.0	4,924,282	74.8	25.2	239,400	100.0	4,647,012	81.3	18.6
Measles-2			-	-	-					-	292,492	97.9	2,870,850	43.6	54.3	239,371	100.0	3,562,158	62.3	37.6
FIC-M1 (without PCV)	-	-	-	-	-	337,735	91.1	3,217,929	43.7	47.4	275,289	92.1	3,882,806	58.9	33.2	217,526	90.8	3,597,523	63.0	27.9
FIC-M1 (with PCV)	-	-	-	-	-	337,704	91.1	3,215,861	43.7	47.4	275,259	92.1	3,880,353	58.9	33.2	217,501	90.8	3,595,095	62.9	27.9
FIC-M1 (with Rota)	-	-	-	-	-	332,609	89.8	3,056,236	41.5	48.2	270,695	90.6	3,658,846	55.5	35.1	213,654	89.2	3,365,367	58.9	30.3
FIC-M2 (without PCV)	-	-	-	-	-	-	-	-	-	-	269,404	90.2	2,419,314	36.7	53.4	217,499	90.8	2,896,691	50.7	40.1
FIC-M2 (with PCV)	-	-	-	-	-	-	-	-	-	-	269,375	90.2	2,418,189	36.7	53.5	217,474	90.8	2,895,013	50.7	40.1
FIC-M2 (with Rota)	-	-	-	-	-	-	-	-	-	-	264,908	88.7	2,298,038	34.9	53.8	213,627	89.2	2,725,456	47.7	41.5

FIC-M1: (1) Without PCV = BCG + OPV1-3 + Penta1-3 + M1, (2) With PCV = BCG + OPV1-3 + Penta1-3 + PCV1-3 + M1 (3), With Rota = BCG + OPV1-3 + Penta1-3 + PCV1-3 + Rota1-2 + M1; FIC-M2: (1) Without PCV = BCG + OPV1-3 + Penta1-3 + M1-2 (2) With PCV = BCG + OPV1-3 + Penta1-3 + PCV1-3 + M1-2, (3) With Rota = BCG + OPV1-3 + Penta1-3 + PCV1-3 + Rota1-2 + M1-2.

**Table 4:** Distribution of defaulters and their coverage among children from 2018 to 2023 birth cohorts enrolled in ZM-EIR across Sindh, Pakistan by various periods—31 January 2018–31 May 2024.

Periods		Defaulters		Coverage Status					
	Total Due			Fully Covered Defaulters		Partially Covered Defaulters		Uncovered Defaulters	
	*n*	*n*	%	*n*	%	*n*	%	*n*	%
Pre-1st lockdown (1 January 2018–22 March 2020)	1,001,803	958,287	95.66	225,115	23.49	569,603	59.44	163,569	17.07
1st national lockdown (23 March–9 May 2020)	794,645	767,891	96.63	236,656	30.82	452,945	58.99	78,290	10.20
Post-1st lockdown (10 May–18 June 2020)	292,520	282,137	96.45	84,522	29.96	166,588	59.05	31,027	11.00
Smart lockdown (19 June–9 August 2020)	675,805	649,408	96.09	190,539	29.34	397,387	61.19	61,482	9.47
Post-smart lockdown (10 August 2020–8 May 2021)	1,002,850	957,190	95.45	360,810	37.69	502,943	52.54	93,437	9.76
2nd lockdown (9–16 May 2021)	120,872	117,801	97.46	39,569	33.59	71,451	60.65	6781	5.76
Restrictions (17 May–6 June 2021)	326,215	315,687	96.77	107,242	33.97	188,637	59.75	19,808	6.27
Post-restrictions (7 June–31 July 2021)	182,397	175,743	96.35	65,128	37.06	100,712	57.31	9903	5.63
3rd lockdown (1–8 August 2021)	120,094	114,856	95.64	44,079	38.38	64,200	55.90	6577	5.73
Post-3rd lockdown 9 August 2021–30 June 2022)	822,703	787,918	95.77	307,842	39.07	437,818	55.57	42,258	5.36
Floods (1 July–31 August 2022)	1,044,658	995,875	95.33	331,506	33.29	607,684	61.02	56,685	5.69
Post-flood (1 September–31 December 2022)	652,128	604,522	92.70	258,841	42.82	304,120	50.31	41,561	6.88
Pandemic last duration (1 January–5 May 2023)	582,728	533,155	91.49	223,676	41.95	274,073	51.41	35,406	6.64
Post-pandemic (6 May 2023–19 March 2024)	1,172,884	966,562	82.41	388,648	40.21	472,503	48.88	105,411	10.91
Total	8,792,302	8,227,032	93.57	2,864,173	34.81	4,610,664	56.04	752,195	9.14

**Table 5:** Predictors of uncovered defaulters vs. immunized children among children from 2018 to 2023 birth cohorts enrolled in ZM-EIR in Sindh province—1 January 2018–31 May 2024.

Predictor	Multivariable Analysis							
	Uncovered Defaulters vs. Immunized Children (*n* = 8,792,392)				Uncovered Defaulters vs. Immunized Children (*n* = 1,434,185)			
	OR	SE	Confidence Interval		OR	SE	Confidence Interval	
**Sex**								
Female	0.99 ***	0.002	0.99	1.00	0.99 **	0.006	0.98	1.00
Male	Ref				Ref			
**Place of Birth**								
Maternity Home	0.72 ***	0.007	0.71	0.73	0.81 ***	0.010	0.79	0.83
Home	0.54 ***	0.003	0.53	0.54	0.83 ***	0.006	0.82	0.84
Hospital	Ref				Ref			
**Residential area**								
Remote Rural	0.60 ***	0.006	0.59	0.62	0.80 ***	0.014	0.78	0.83
Urban	2.61 ***	0.008	2.59	2.62	1.54 ***	0.011	1.52	1.56
Non-Remote Rural	Ref				Ref			
**Enrollment modality**								
Fixed	3.17 ***	0.010	3.15	3.19	2.11 ***	0.018	2.08	2.15
EOA	0.86 ***	0.005	0.85	0.87	0.69 ***	0.013	0.67	0.72
Van	1.09 ***	0.019	1.05	1.13	1.41 **	0.162	1.13	1.77
Outreach	Ref				Ref			
**Mother’s Education (in years)**								
0	0.36 ***	0.004	0.35	0.37	0.72 ***	0.009	0.71	0.74
1–5	0.43 ***	0.005	0.42	0.44	0.69 ***	0.008	0.68	0.71
6–8	0.60 ***	0.008	0.59	0.62	0.72 ***	0.011	0.70	0.74
9–10	0.83 ***	0.011	0.81	0.85	0.78 ***	0.011	0.76	0.81
≥11	Ref				Ref			
**Enrollment Age (months)**								
0–1	0.59 ***	0.005	0.58	0.60	0.83 ***	0.051	0.74	0.94
2–3	0.35 ***	0.003	0.34	0.35	0.46 ***	0.028	0.40	0.51
4–6	0.11 ***	0.001	0.11	0.11	0.13 ***	0.009	0.12	0.15
7–9	0.23 ***	0.003	0.23	0.24	0.27 ***	0.017	0.23	0.30
10–12	0.09 ***	0.001	0.09	0.09	0.09 ***	0.007	0.08	0.10
13–15	0.99	0.011	0.97	1.01	0.04 ***	0.006	0.03	0.06
16–18	0.18 ***	0.003	0.18	0.19	0.01 ***	0.004	0.01	0.03
>18	Ref				Ref			
**Provision of Contact Number**								
Not provided	0.62 ***	0.001	0.61	0.62	0.85 ***	0.007	0.84	0.87
Provided	Ref				Ref			
**Provision of CNIC Numbers**								
Not provided	0.60 ***	0.002	0.60	0.61	1.03 **	0.008	1.01	1.04
Provided	Ref				Ref			
**SMS Reminders**								
Not opted	0.64 ***	0.002	0.64	0.65	1.27 ***	0.012	1.25	1.29
Opted	Ref				Ref			
**Birth Year**								
2019	0.67 ***	0.003	0.67	0.68	0.57 ***	0.005	0.57	0.58
2020	0.43 ***	0.002	0.43	0.43	0.40 ***	0.004	0.39	0.41
2021	0.29 ***	0.001	0.29	0.29	0.25 ***	0.003	0.24	0.25
2022	0.31 ***	0.001	0.31	0.32	0.25 ***	0.003	0.24	0.25
2023	0.45 ***	0.002	0.45	0.45	0.34 ***	0.005	0.33	0.34
2018	Ref				Ref			

** *p* < 0.01, *** *p* < 0.001 indicate statistical significance.

## Data Availability

Data may be obtained from a third party and are not publicly available. The data used for this analysis from the Sindh Electronic Immunization Registry (SEIR; also known as the Zindagi Mehfooz program; ZM-EIR) can be requested from the Government of Sindh’s Expanded Programme on Immunization (EPI).

## Supplementary Materials

The following supporting information can be downloaded at: https://www.mdpi.com/article/10.3390/vaccines12121327/s1, Table S1: EPI recommended vaccination age and timeliness; Table S2: Time interval between the administration of subsequent doses among children from 2018 to 2023 birth cohorts enrolled in ZM-EIR across Sindh, Pakistan by birth cohort (*n* = 8,792,329) 1 January 2018–31 May 2024; Table S3.1: Antigen-wise age-appropriate coverage rates at 6 months among children from 2018 to 2023 birth cohorts enrolled in ZM-EIR across Sindh, Pakistan by birth cohort and defaulter status; Table S3.2: Antigen-wise age-appropriate coverage rates at 12 months among children from 2018 to 2023 birth cohorts enrolled in ZM-EIR across Sindh, Pakistan by birth cohort and defaulter status; Table S3.3: Antigen-wise age-appropriate coverage rates at 18 months among children from 2018 to 2023 birth cohorts enrolled in ZM-EIR across Sindh, Pakistan by birth cohort and defaulter status; Table S3.4: Antigen-wise age-appropriate coverage rates at 23 months among children from 2018 to 2023 birth cohorts enrolled in ZM-EIR across Sindh, Pakistan by birth cohort and defaulter status; Figure S1: Dropout rates among children from 2018–2023 birth cohorts enrolled in ZM-EIR across Sindh, Pakistan by birth cohort; Figure S2: Antigen-wise age-appropriate coverage rates at 6, 12, 18, and 23 months among children from 2018 to 2023 birth cohorts enrolled in ZM-EIR across Sindh, Pakistan by birth cohort (*n* = 8,792,329); Figure S3: Kaplan–Meier curves for three doses of pentavalent vaccines among children from 2018–2023 birth cohorts enrolled in ZM-EIR across Sindh, Pakistan (*n* = 8,792,329) by birth year; Figure S4: Antigen-wise comparison of immunization doses administered through different modalities among children from 2018 to 2023 birth cohorts enrolled in ZM-EIR across Sindh, Pakistan (*n* = 8,792,329).
