# Supplementary material for: Leveraging Data from a Provincial Electronic Immunization Registry to Analyze Immunization Coverage, Timeliness, and Defaulters Among 8.8 Million Children from the 2018 to 2023 Birth Cohorts in Sindh Province, Pakistan
Source: Vaccines (Basel). 2024 Nov 26;12(12):1327. doi: 10.3390/vaccines12121327 (PMC11679383; doi:10.3390/vaccines12121327)
Supplement: Supplementary file 1 [file vaccines-12-01327-s001.zip › vaccines-3300826-supplementary.pdf]

Supplementary Material: Tables and Figures

Tables

Table S1: EPI recommended vaccination age and timeliness

#	Vaccine	Validity as per EPI-Pakistan schedule (age)	Timeliness (days)	Coverage (n)	Lost to follow-up
1.	BCG	0-365 days	0-28 days	Validity(n)/Children > 28days	Children who have not received BCG till 28 days after the scheduled date has passed and have not returned for any vaccine past the scheduled date
2.	OPV -0	0-28 days	0-28 days	Validity(n)/Children > 28days	Children who have not received OPV-0 till 28 days after the scheduled date has passed and have not returned for any vaccine past the scheduled date
3.	HepatitisB-0 **(optional)**	0-3 days	0-3 days	Validity(n)/Children > 3days	Children who have not received HepatitisB-0 till 3 days after the scheduled date has passed and have not returned for any vaccine past the scheduled date
4.	Penta-1/OPV-1/PCV-1/rota-1	>=42 days	39-70 days	Validity(n)/Children >42days	Children who have not received OPV-1 till 28 days after the scheduled date has passed and have not returned for any vaccine past the scheduled date
5.	Penat-2/OPV-2/PCV-2/Rota-2	>=4 weeks after OPV-1 & age >= 67 days	67-98 days	Validity(n)/Children>67 days and those having received OPV-1	Children who have not received OPV-2 till 28 days after the scheduled date has passed and have not returned for any vaccine past the scheduled date

6.	Penta-3/OPV-3/PCV-3/IPV-1	>= 4 weeks after OPV-2 & age>= 95 days	95-126 days	Validity(n)/Children>95days and those having received OPV-2	Children who have not received OPV-3 till 28 days after the scheduled date has passed and have not returned for any vaccine past the scheduled date
7.	Measles1/IPV-2/TCV	>39weeks	270-301days	Validity(n)/Children>279days	Children who have not received Measles1 till 28 days after the scheduled date has passed and have not returned for any vaccine past the scheduled date
8.	Measles2	>52weeks &>4 weeks from Measles 1	453-484 days	Validity(n)/Children453>days and those having received Measles-1	Children who have not received Measles2 till 28 days after the scheduled date has passed and have not returned for any vaccine past the scheduled date

Table S2: Time interval between the administration of subsequent doses among children from 2018-2023 birth cohorts enrolled in ZM-EIR across Sindh, Pakistan by birth cohort (n=8,792,329) Jan 1, 2018-May 31, 2024

	**2018 (n= 1,025,904)**				**2019 (n= 1,349,301)**				**2020 (n= 1,427,321)**				**2021 (n= 1,456,235)**				**2022 (n= 1,781,011)**			
	**Total**	**Invalid doses**	**Median (IQR)**	**Range**	**Total**	**Invalid doses**	**Median (IQR)**	**Range**	**Total**	**Invalid doses**	**Median (IQR)**	**Range**	**Total**	**Invalid doses**	**Median (IQR)**	**Range**	**Total**	**Invalid doses**	**Median (IQR)**	**Range**
Penta-1 to Penta-2	669,808	4,897	40 (32 - 68)	1 - 706	947,019	4,755	42 (32 - 78)	1 - 707	1,098,232	3,730	43 (32 - 82)	1 - 714	1,158,992	4,628	50 (34 - 84)	1 - 728	1,485,420	3,751	45 (32 - 77)	1 - 714
Penta-2 to Penta-3	608,239	3,778	42 (32 - 80)	1 - 684	883,904	3,200	46 (33 - 91)	1 - 680	1,064,069	2,893	48 (33 - 99)	1 - 687	1,146,811	2,376	53 (34 - 87)	1 - 709	1,436,282	1,898	46 (33 - 79)	1 - 682
Penta-1 to Penta-3	600,413	1,129	92 (68 - 160)	1 - 728	869,983	832	101 (70 - 195)	1 - 728	1,057,278	707	110 (71 - 199)	1 - 727	1,144,122	777	115 (77 - 180)	1 - 718	1,459,300	545	102 (71 - 160)	1 - 722
PCV-1 to PCV-2	669,821	4,523	40 (32 - 68)	1 - 706	946,450	4,405	42 (32 - 78)	1 - 707	1,097,487	3,577	43 (32 - 82)	1 - 714	1,158,607	4,401	50 (34 - 84)	1 - 728	1,485,161	3,626	45 (32 - 77)	1 - 714
PCV-2 to PCV-3	608,399	3,435	42 (32 - 80)	1 - 700	883,279	2,913	46 (33 - 91)	1 - 681	1,063,382	2,680	48 (33 - 99)	1 - 687	1,146,309	2,232	53 (34 - 87)	1 - 709	1,435,825	1,831	46 (33 - 79)	1 - 682

PCV-1 to PCV-3	600,572	1,189	92 (68 - 160)	1 - 728	869,431	844	101 (70 - 195)	1 - 728	1,056,534	710	110 (71 - 199)	1 - 727	1,143,570	786	115 (77 - 180)	1 - 718	1,458,823	551	102 (71 - 160)	1 - 722
OPV-1 to OPV-2	668,810	4,793	40 (32 - 68)	1 - 705	945,336	4,682	42 (32 - 78)	1 - 707	1,098,560	3,616	43 (32 - 83)	1 - 714	1,159,172	4,429	50 (34 - 84)	1 - 728	1,484,426	3,614	45 (32 - 77)	1 - 714
OPV-2 to OPV-3	607,912	3,480	42 (32 - 80)	1 - 700	883,171	2,942	46 (33 - 91)	1 - 705	1,064,489	2,686	48 (33 - 99)	1 - 687	1,146,479	2,235	53 (34 - 88)	1 - 713	1,435,100	1,900	46 (33 - 79)	1 - 682
OPV-1 to OPV-3	599,696	1,191	92 (68 - 160)	1 - 728	868,673	879	101 (70 - 195)	1 - 728	1,057,391	713	110 (71 - 199)	1 - 727	1,143,846	791	115 (77 - 180)	1 - 718	1,457,972	547	102 (71 - 160)	1 - 722
Rota-1 to Rota-2	593,216	4,738	40 (32 - 68)	1 - 706	912,261	4,631	42 (32 - 77)	1 - 707	1,065,724	3,911	43 (32 - 82)	1 - 714	1,134,974	4,769	50 (34 - 84)	1 - 728	1,471,857	4,442	45 (32 - 77)	1 - 714
IPV-1 to IPV-2	45	11	157 (107 - 246)	21 - 602	48,982	156	353 (252 - 466)	1 - 721	670,367	1,484	201 (141 - 327)	1 - 696	1,002,226	1,490	155 (103 - 203)	1 - 659	1,257,809	1,475	159 (114 - 195)	1 - 661
Measles-1 to Measles-2	434,480	92	196 (173 - 245)	1 - 725	713,798	65	179 (118 - 221)	1 - 727	897,642	79	182 (126 - 223)	1 - 720	1,051,676	45	182 (140 - 217)	1 - 692	1,134,181	32	182 (149 - 211)	1 - 721

Invalid doses: The vaccination date of the booster vaccine is less than or equal to the initial dose. For instance Pneta2 vaccination date<=Penta1 vaccination date

Table S3.1: Antigen-wise age-appropriate coverage rates at 6 months among children from 2018-2023 birth cohorts enrolled in ZM-EIR across Sindh, Pakistan by birth cohort and defaulter status

	6 months																	
	2018 (n=1,025,904)			2019 (n=1,349,301)			2020 (n=1,427,321)			2021 (n=1,456,235)			2022 (n=1,781,011)			2023 (n=1,127,964)		
	Non-Defaulter (n=44,430)	Defaulter (n=9,814,74)	Percentage difference	Non-Defaulter (n=56,217)	Defaulter (n=1,293,084)	Percentage difference	Non-Defaulter (n=54,663)	Defaulter (n=1,372,658)	Percentage difference	Non-Defaulter (n=50,869)	Defaulter (n=1,405,366)	Percentage difference	Non-Defaulter (n=1,037,92)	Defaulter (n=1,677,219)	Percentage difference	Non-Defaulter (n=1,037,55)	Defaulter (n=1,024,209)	Percentage difference
	%	%	%	%	%	%	%	%	%	%	%	%	%	%	%	%	%	%
BCG	89.6	74.0	15.6	93.9	75.9	18.0	97.5	78.7	18.8	96.7	77.6	19.1	98.7	80.0	18.7	98.7	84.8	13.9
Penta-1	88.9	66.8	22.0	94.6	70.7	23.9	97.7	73.3	24.4	96.7	73.5	23.2	99.1	78.5	20.6	92.7	82.2	10.4
OPV-1	88.9	66.6	22.3	94.6	70.6	24.1	97.7	73.4	24.3	96.7	73.6	23.1	99.1	78.6	20.5	92.6	82.3	10.3
PCV-1	88.9	66.8	22.0	94.6	70.7	23.9	97.7	73.3	24.4	96.7	73.5	23.2	99.1	78.5	20.6	92.7	82.2	10.4
Rota-1	87.3	61.5	25.9	94.9	70.9	24.0	97.8	73.5	24.3	96.8	74.0	22.8	99.1	78.6	20.5	92.8	82.8	9.9

Penta-2	87.2	50.0	37.2	94.0	52.4	41.7	96.8	54.1	42.7	96.2	54.2	41.9	98.9	60.1	38.8	88.7	63.5	25.2
OPV-2	87.2	50.0	37.2	94.0	52.3	41.7	96.9	54.1	42.7	96.2	54.3	41.9	98.8	60.1	38.8	88.7	63.5	25.2
PCV-2	87.2	50.0	37.2	94.0	52.4	41.7	96.9	54.1	42.8	96.2	54.2	41.9	98.9	60.0	38.8	88.7	63.5	25.2
Rota-2	84.1	43.1	41.0	92.9	50.0	42.9	95.4	51.8	43.6	94.3	52.2	42.1	97.7	58.2	39.4	87.4	62.0	25.4
Penta-3	85.6	32.1	53.5	93.2	32.7	60.5	94.8	32.6	62.2	95.8	30.9	64.9	98.4	37.6	60.8	85.5	38.9	46.6
OPV-3	85.6	32.0	53.6	93.2	32.7	60.5	94.8	32.6	62.2	95.8	30.9	64.9	98.4	37.6	60.9	85.4	38.9	46.6
PCV-3	85.6	32.1	53.5	93.2	32.7	60.5	94.8	32.6	62.2	95.8	30.9	64.9	98.4	37.6	60.9	85.5	38.9	46.6
IPV-1	85.6	35.5	50.1	93.1	37.3	55.8	94.7	36.6	58.2	95.8	37.1	58.7	98.4	43.4	55.0	85.4	45.5	40.0

Table S3.2: Antigen-wise age-appropriate coverage rates at 12 months among children from 2018-2023 birth cohorts enrolled in ZM-EIR across Sindh, Pakistan by birth cohort and defaulter status

	**12 months**					
	**2018 (n=1,025,904)**	**2019 (n=1,349,301)**	**2020 (n=1,427,321)**	**2021 (n=1,456,235)**	**2022 (n=1,781,011)**	**2023 (n=1,851,28)**

	Non-Defaulter (n=44,430)	Defaulter (n=9,814,74)	Percentage difference	Non-Defaulter (n=56,217)	Defaulter (n=1,293,084)	Percentage difference	Non-Defaulter (n=54,663)	Defaulter (n=1,372,658)	Percentage difference	Non-Defaulter (n=50,869)	Defaulter (n=1,405,366)	Percentage difference	Non-Defaulter (n=1,037,92)	Defaulter (n=1,677,219)	Percentage difference	Non-Defaulter (n=60,618)	Defaulter (n=629,463)	Percentage difference
	%	%	%	%	%	%	%	%	%	%	%	%	%	%	%	%	%	%
BCG	89.6	79.9	9.7	93.9	80.8	13.0	97.5	85.3	12.1	96.7	84.9	11.8	98.7	88.6	10.1	99.0	89.9	9.2
Penta-1	88.9	75.9	12.9	94.6	79.5	15.1	97.7	85.0	12.7	96.7	86.4	10.3	99.1	90.0	9.0	99.2	91.2	8.0
OPV-1	88.9	75.7	13.1	94.6	79.4	15.2	97.7	85.1	12.6	96.7	86.4	10.2	99.1	90.1	9.0	99.2	91.2	7.9
PCV-1	88.9	75.9	12.9	94.6	79.5	15.1	97.7	85.0	12.7	96.7	86.3	10.3	99.1	90.0	9.1	99.2	91.2	8.0
Rota-1	87.3	70.4	16.9	94.9	79.4	15.5	97.8	84.9	13.0	96.8	86.1	10.6	99.1	90.0	9.1	99.2	91.5	7.7
Penta-2	87.2	63.4	23.8	94.0	67.1	27.0	96.8	73.9	22.9	96.2	76.7	19.5	98.9	80.7	18.1	99.0	83.4	15.6
OPV-2	87.2	63.4	23.8	94.0	67.0	27.0	96.9	74.0	22.9	96.2	76.7	19.5	98.8	80.8	18.1	99.0	83.4	15.6
PCV-2	87.2	63.5	23.7	94.0	67.1	27.0	96.9	73.9	22.9	96.2	76.7	19.5	98.9	80.7	18.1	99.0	83.4	15.6
Rota-2	84.1	55.1	29.1	92.9	63.3	29.6	95.4	70.1	25.3	94.3	73.1	21.2	97.7	77.6	20.1	97.8	80.5	17.3
Penta-3	85.6	53.1	32.5	93.2	55.4	37.8	94.8	62.2	32.6	95.8	68.0	27.8	98.4	72.5	26.0	98.8	77.4	21.4

OPV-3	85.6	53.1	32.5	93.2	55.4	37.8	94.8	62.2	32.6	95.8	68.0	27.8	98.4	72.4	26.0	98.7	77.4	21.3
PCV-3	85.6	53.2	32.4	93.2	55.4	37.8	94.8	62.2	32.7	95.8	68.0	27.8	98.4	72.4	26.0	98.8	77.4	21.4
IPV-1	85.6	57.8	27.8	93.1	60.1	33.1	96.3	67.4	28.9	97.7	75.2	22.5	99.4	78.7	20.7	99.8	82.6	17.2
Measles-1	89.5	46.2	43.3	94.6	44.3	50.3	96.2	50.5	45.7	97.2	61.8	35.4			0.0	93.5	66.6	26.9
FIC-M1 (without PCV)	79.8	33.8	46.0	87.8	32.9	54.8	91.2	39.6	51.6	92.8	48.5	44.2	96.7	52.4	44.4	91.5	56.5	35.0
FIC-M1 (with PCV)	79.8	33.8	46.1	87.8	32.9	54.9	91.2	39.6	51.6	92.7	48.5	44.2	96.7	52.3	44.4	91.5	56.5	35.0
FIC-M1 (with Rota)	77.3	29.6	47.7	86.9	31.2	55.7	89.9	37.6	52.2	91.0	46.4	44.6	95.6	50.6	45.0	90.4	54.8	35.6

Table S3.3: Antigen-wise age-appropriate coverage rates at 18 months among children from 2018-2023 birth cohorts enrolled in ZM-EIR across Sindh, Pakistan by birth cohort and defaulter status

	18 months														
	2018 (n=1,025,904)			2019 (n=1,349,301)			2020 (n=1,427,321)			2021 (n=1,456,235)			2022 (n=9,396,68)		
	Non-Defaulter (n=44,430) %	Defaulter (n=9,814,74) %	Percentage difference %	Non-Defaulter (n=56,217) %	Defaulter (n=1,293,084) %	Percentage difference %	Non-Defaulter (n=54,663) %	Defaulter (n=1,372,658) %	Percentage difference %	Non-Defaulter (n=50,869) %	Defaulter (n=1,405,366) %	Percentage difference %	Non-Defaulter (n=92,577) %	Defaulter (n=1,534,470) %	Percentage difference %
BCG	89.6	81.1	8.5	93.9	81.8	12.0	97.5	86.2	11.3	96.7	86.1	10.5	98.7	89.5	9.2
Penta-1	88.9	78.9	10.0	94.6	82.7	11.9	97.7	87.5	10.2	96.7	89.5	7.1	99.1	92.2	6.8
OPV-1	88.9	78.6	10.2	94.6	82.6	12.0	97.7	87.6	10.1	96.7	89.6	7.1	99.1	92.3	6.8
PCV-1	88.9	78.8	10.0	94.6	82.7	11.9	97.7	87.5	10.2	96.7	89.5	7.1	99.1	92.2	6.9
Rota-1	87.3	73.2	14.2	94.9	82.4	12.5	97.8	87.1	10.7	96.8	89.1	7.7	99.0	92.1	6.9
Penta-2	87.2	68.2	19.0	94.0	73.7	20.3	96.8	79.6	17.2	96.2	83.5	12.7	98.8	86.4	12.4
OPV-2	87.2	68.1	19.1	94.0	73.7	20.4	96.9	79.7	17.2	96.2	83.5	12.6	98.8	86.5	12.3
PCV-2	87.2	68.2	19.0	94.0	73.7	20.4	96.9	79.6	17.2	96.2	83.5	12.7	98.8	86.4	12.4
Rota-2	84.1	59.1	25.0	92.9	69.1	23.8	95.4	75.2	20.2	94.3	79.1	15.2	97.6	82.8	14.8
Penta-3	85.6	60.6	25.0	93.2	66.1	27.1	94.8	72.8	22.0	95.8	80.1	15.7	98.4	82.4	16.0

OPV-3	85.6	60.6	25.0	93.2	66.1	27.1	94.8	72.9	22.0	95.8	80.1	15.7	98.4	82.4	16.0
PCV-3	85.6	60.7	25.0	93.2	66.1	27.1	94.8	72.8	22.0	95.8	80.1	15.7	98.4	82.4	16.0
IPV-1	85.6	65.1	20.5	93.1	69.7	23.4	97.1	78.3	18.8	98.4	86.5	11.9	99.5	87.4	12.0
Measles-1	100.0	64.5	35.5	100.0	67.9	32.0	99.9	72.5	27.4	100.0	82.7	17.3	100.0	81.8	18.2
Measles-2	100.0	29.0	71.0	100.0	36.4	63.5	99.9	41.7	58.3	100.0	53.6	46.4	93.3	51.5	41.8
FIC-M1 (with out PCV)	81.7	45.2	36.5	89.5	49.5	40.0	93.2	57.8	35.3	93.6	66.5	27.1	97.3	69.7	27.6
FIC-M1 (with PCV)	81.7	45.2	36.5	89.5	49.4	40.1	93.2	57.8	35.4	93.6	66.5	27.1	97.3	69.7	27.6
FIC-M1 (with Rota)	79.0	39.6	39.4	88.5	46.5	42.0	91.7	54.7	37.0	91.8	63.2	28.6	96.1	67.1	29.1
FIC-M2 (with out PCV)	81.7	21.7	59.9	89.5	28.4	61.1	93.2	35.5	57.6	93.6	45.6	48.0	91.0	46.2	44.8
FIC-M2 (with PCV)	81.7	21.7	59.9	89.5	28.4	61.1	93.1	35.5	57.6	93.6	45.6	48.0	91.0	46.2	44.8
FIC-M2	78.9	18.9	60.0	88.5	26.9	61.7	91.7	33.7	58.0	91.8	43.6	48.2	89.9	44.9	45.0

(with
Rota)

Table S3.4: Antigen-wise age-appropriate coverage rates at 23 months among children from 2018-2023 birth cohorts enrolled in ZM-EIR across Sindh, Pakistan by birth cohort and defaulter status

	**23 months**														
	**2018 (n=1,025,904)**			**2019 (n=1,349,301)**			**2020 (n=1,427,321)**			**2021 (n=1,456,235)**			**2022 (n=9,396,68)**		
	**Non-Defaulter (n=44,430)**	**Defaulter (n=9,814,74)**	**Percentage difference**	**Non-Defaulter (n=56,217)**	**Defaulter (n=1,293,084)**	**Percentage difference**	**Non-Defaulter (n=54,663)**	**Defaulter (n=1,372,658)**	**Percentage difference**	**Non-Defaulter (n=50,869)**	**Defaulter (n=1,405,366)**	**Percentage difference**	**Non-Defaulter (n=92,577)**	**Defaulter (n=1,534,470)**	**Percentage difference**
	%	%	%	%	%	%	%	%	%	%	%	%	%	%	%
BCG	89.6	81.4	8.2	93.9	82.2	11.7	97.5	86.6	10.9	96.7	86.5	10.2	98.4	88.6	9.8
Penta-1	88.9	79.7	9.2	94.6	83.8	10.8	97.7	89.0	8.7	96.7	90.6	6.1	98.9	92.6	6.3
OPV-1	88.9	79.5	9.4	94.6	83.7	10.9	97.7	89.1	8.6	96.7	90.7	6.0	98.9	92.7	6.2
PCV-1	88.9	79.7	9.2	94.6	83.7	10.9	97.7	89.0	8.7	96.7	90.6	6.1	98.9	92.6	6.3
Rota-1	87.3	74.0	13.3	94.9	83.3	11.6	97.8	88.5	9.3	96.8	90.2	6.6	98.9	92.4	6.5
Penta-2	87.2	69.9	17.3	94.0	76.0	18.0	96.8	82.9	13.9	96.2	85.9	10.2	98.7	88.0	10.7

OPV-2	87.2	69.9	17.3	94.0	76.0	18.0	96.9	82.9	14.0	96.2	86.0	10.2	98.7	88.0	10.7
PCV-2	87.2	69.9	17.3	94.0	76.0	18.0	96.9	82.9	14.0	96.2	85.9	10.2	98.7	88.0	10.7
Rota-2	84.1	60.5	23.6	92.9	71.1	21.8	95.4	77.9	17.5	94.3	81.2	13.1	97.5	84.0	13.6
Penta-3	85.6	63.6	22.0	93.2	70.4	22.8	94.8	79.5	15.3	95.8	84.4	11.4	98.3	85.8	12.5
OPV-3	85.6	63.6	22.0	93.2	70.4	22.8	94.8	79.5	15.3	95.8	84.4	11.4	98.3	85.7	12.6
PCV-3	85.6	63.7	21.9	93.2	70.4	22.8	94.8	79.5	15.3	95.8	84.4	11.4	98.3	85.8	12.5
IPV-1	85.6	67.7	17.9	93.2	73.8	19.4	97.1	85.0	12.1	98.4	90.3	8.1	99.4	90.6	8.8
Measles-1	100.0	70.9	29.1	100.0	75.7	24.3	99.9	83.8	16.1	100.0	88.6	11.4	100.0	87.4	12.6
Measles-2	100.0	45.9	54.1	100.0	55.7	44.3	99.9	64.4	35.5	100.0	73.5	26.5	100.0	71.9	28.1
FIC-M1 (without PCV)	81.7	48.9	32.8	89.5	54.7	34.8	93.2	66.8	26.4	93.6	71.5	22.1	97.2	73.9	23.3
FIC-M1 (with PCV)	81.7	48.9	32.8	89.5	54.7	34.8	93.2	66.8	26.4	93.6	71.4	22.2	97.2	73.8	23.3

FIC-M1 (with Rota)	79.0	42.7	36.3	88.5	51.3	37.2	91.7	62.9	28.8	91.8	67.7	24.1	96.0	70.8	25.2
FIC-M2 (with out PCV)	81.7	33.5	48.2	89.5	42.4	47.1	93.2	53.8	39.4	93.6	61.6	32.0	97.2	62.9	34.2
FIC-M2 (with PCV)	81.7	33.5	48.2	89.5	42.4	47.1	93.1	53.8	39.3	93.6	61.6	32.1	97.2	62.9	34.3
FIC-M2 (with Rota)	78.9	29.3	49.6	88.5	39.8	48.7	91.7	50.8	40.9	91.8	58.6	33.2	96.0	60.7	35.3

Figures

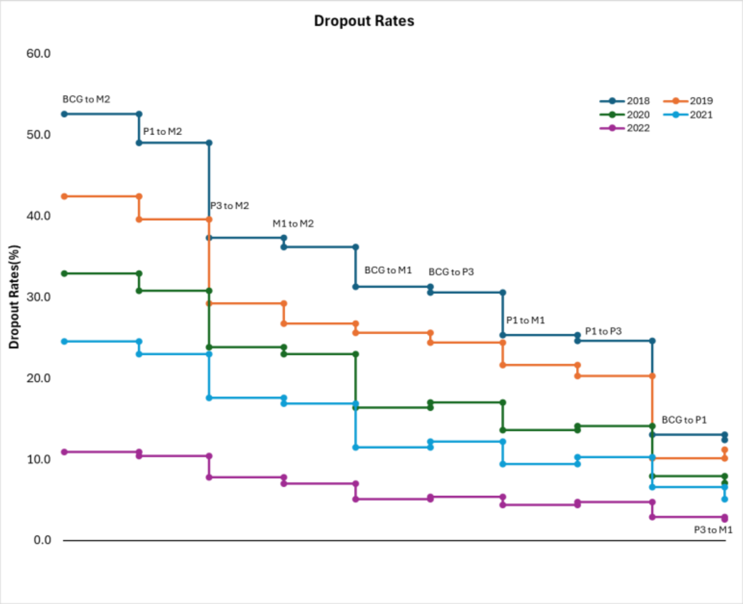

Figure S1. Dropout rates among children from 2018-2023 birth cohorts enrolled in ZM-EIR across Sindh, Pakistan by birth cohort*. * The dropout rate in our study was calculated by dividing the number of children who received the first vaccine minus the number of children who received the last vaccine with the number of children who received the first vaccine multiplied by 100 (((first vaccine - last vaccine) ÷ first vaccine) × 100%)).

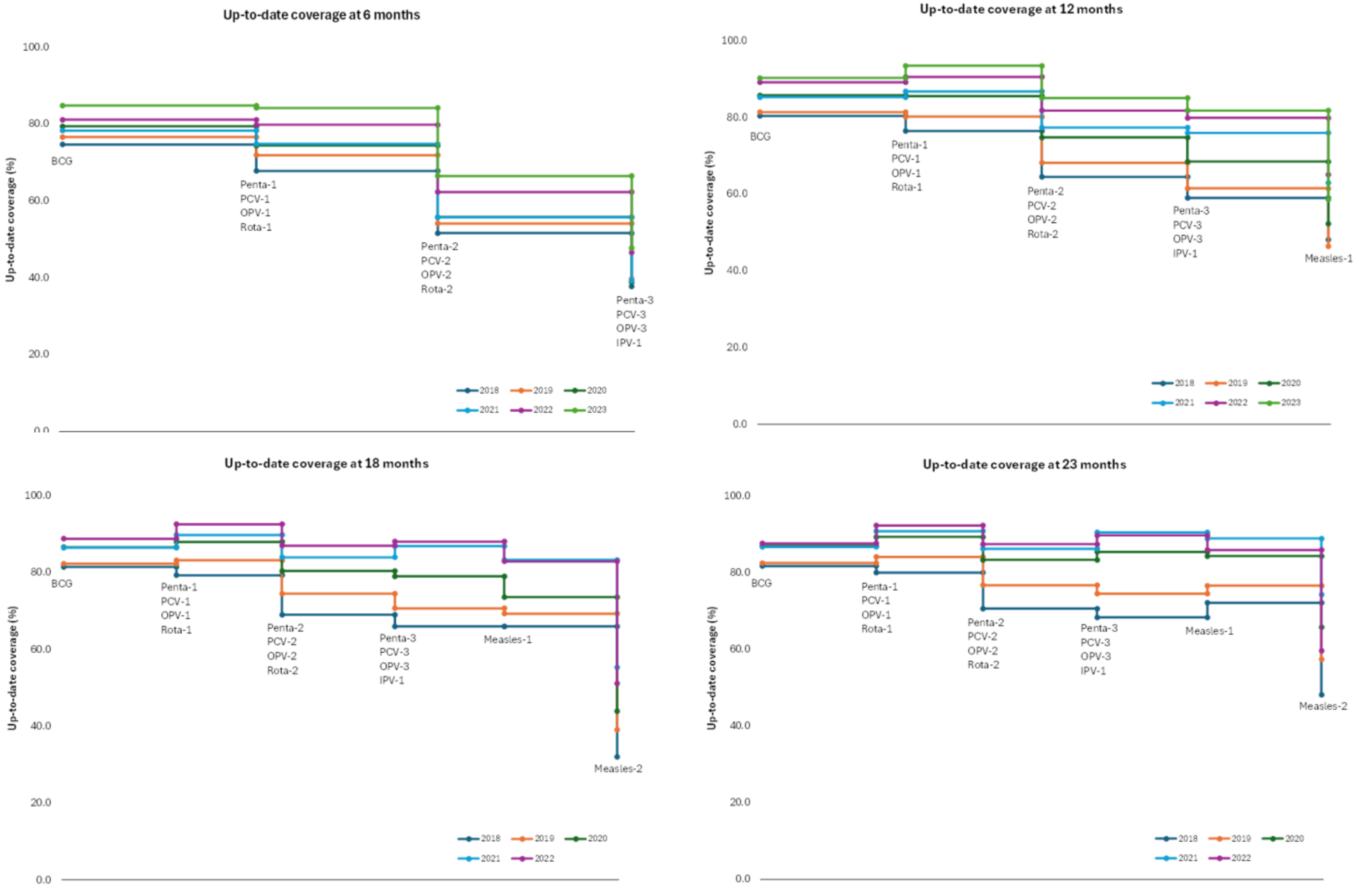

Figure S2. Antigen-wise age-appropriate coverage rates at 6, 12, 18, and 23 months among children from 2018-2023 birth cohorts enrolled in ZM-EIR across Sindh, Pakistan by birth cohort (n=8,792,329).

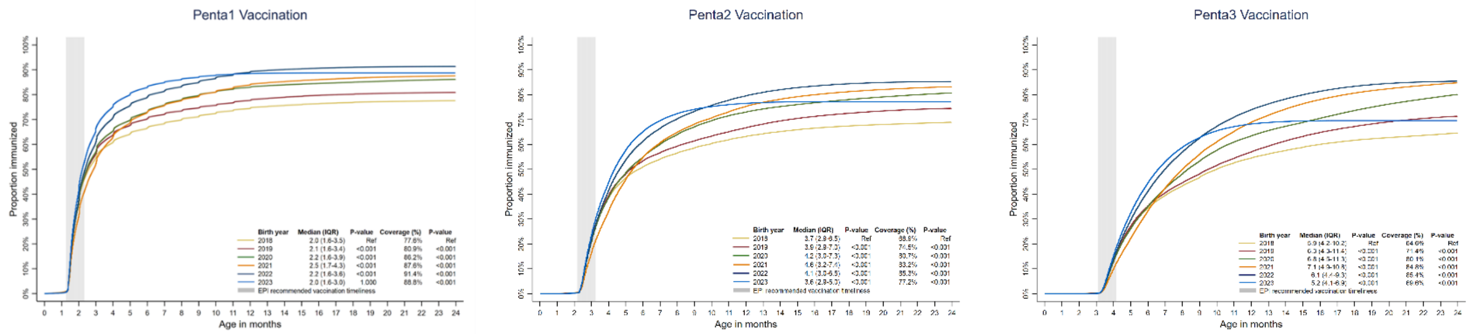

Figure S3: Kaplan-Meier curves for three doses of pentavalent vaccines among children from 2018-2023 birth cohorts enrolled in ZM-EIR across Sindh, Pakistan (n=8,792,329) by birth year

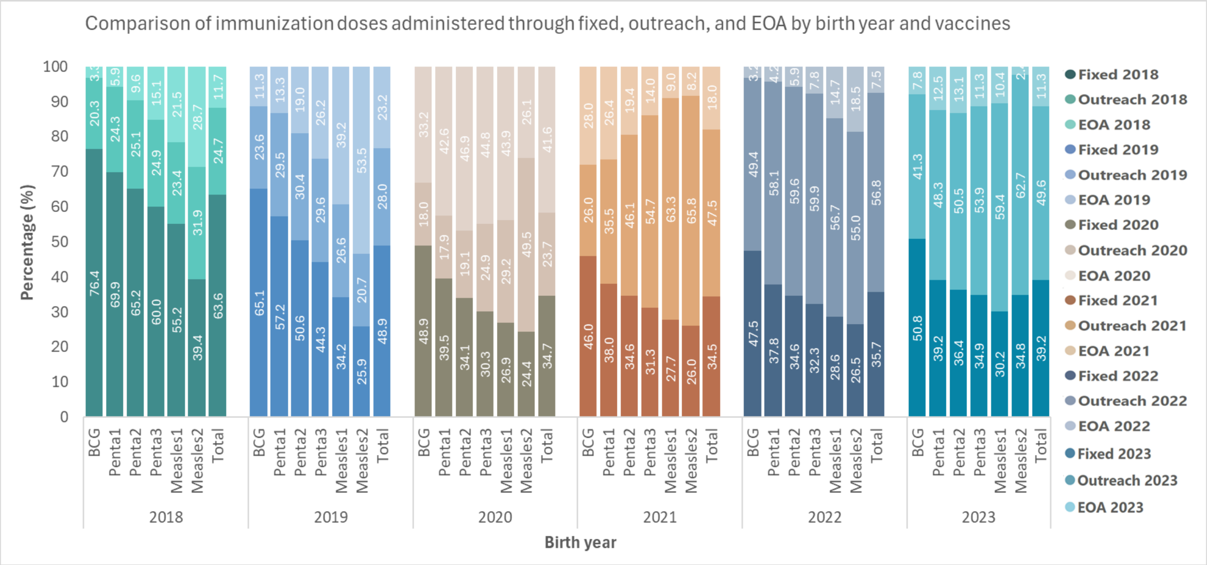

Figure S4: Antigen-wise comparison of immunization doses administered through different modalities among children from 2018-2023 birth cohorts enrolled in ZM-EIR across Sindh, Pakistan (n=8,792,329**).**
